# Genome-wide mapping of FOXM1 binding reveals co-binding with estrogen receptor alpha in breast cancer cells

**DOI:** 10.1186/gb-2013-14-1-r6

**Published:** 2013-01-24

**Authors:** Deborah A Sanders, Caryn S Ross-Innes, Dario Beraldi, Jason S Carroll, Shankar Balasubramanian

**Affiliations:** 1 Cancer Research UK, Cambridge Research Institute, Li Ka Shing Centre, Robinson Way, Cambridge, CB2 0RE, UK; 2 Department of Oncology, University of Cambridge, Cambridge, CB2 0RE, UK; 3 Department of Chemistry, University of Cambridge, Lensfield Road, Cambridge, CB2 1EW, UK; 4 School of Clinical Medicine, The University of Cambridge, Addenbrooke's Hospital, Hills Road, Cambridge CB2 0SP, UK

## Abstract

**Background:**

The forkhead transcription factor FOXM1 is a key regulator of the cell cycle. It is frequently over-expressed in cancer and is emerging as an important therapeutic target. In breast cancer FOXM1 expression is linked with estrogen receptor (ERα) activity and resistance to endocrine therapies, with high levels correlated with poor prognosis. However, the precise role of FOXM1 in ER positive breast cancer is not yet fully understood.

**Results:**

The study utilizes chromatin immunoprecipitation followed by high-throughput sequencing to map FOXM1 binding in both ERα-positive and -negative breast cancer cell lines. The comparison between binding site distributions in the two cell lines uncovered a previously undescribed relationship between binding of FOXM1 and ERα. Further molecular analyses demonstrated that these two factors can bind simultaneously at genomic sites and furthermore that FOXM1 regulates the transcriptional activity of ERα via interaction with the coactivator CARM1. Inhibition of FOXM1 activity using the natural product thiostrepton revealed down-regulation of a set of FOXM1-regulated genes that are correlated with patient outcome in clinical breast cancer samples.

**Conclusions:**

These findings reveal a novel role for FOXM1 in ERα transcriptional activity in breast cancer and uncover a FOXM1-regulated gene signature associated with ER-positive breast cancer patient prognosis.

## Background

The forkhead transcription factor FOXM1 is a key regulator of the cell cycle [1,2] critical for the G1 to S phase transition and G2 to M progression [3]. Expression of FOXM1 is essential for mitotic spindle assembly and correct chromosome segregation with depletion leading to mitotic catastrophe and cell cycle arrest [4]. FOXM1 is also known to regulate the expression of genes involved in angiogenesis [5], metastasis [6] and response to oxidative stress and DNA damage [7,8]. Overexpression of FOXM1 has been reported in many types of cancer [9] and is correlated with poor prognosis [10,11]. Aberrant FOXM1 expression is an early event in oncogenesis [12], possibly acting as an initiating factor [13] and has been associated with genomic instability [12].

Breast cancer is one of the leading causes of cancer mortality in women and numerous studies have shown a correlation between FOXM1 expression and breast cancer progression [4,14,15], suggesting that FOXM1 is a potential prognostic breast tumor marker [16]. FOXM1 expression in breast cancer was found to correlate with levels of YWHAZ, a member of the 14-3-3 family of proteins [17] and also with HER2 status [15,16]. Meta-analysis of gene expression data from breast cancer patient studies identified FOXM1 as one of 117 genes comprising a gene expression signature predictive of survival [18]. FOXM1 over-expression has also been linked with drug resistance in breast cancer chemotherapy [19,20] and therefore poor clinical prognosis.

Approximately 70% of breast cancers are estrogen receptor (ERα)-positive and there is increasing evidence to suggest that ERα and FOXM1 act as co-regulators. FOXM1 and ERα regulate the expression of each other in a positive cross-regulatory loop [21,22]. FOXM1 has previously been identified as an ERα-responsive gene [23] and has been suggested to act as a prognostic marker in endocrine-positive cancers [24]. Furthermore, resistance to anti-estrogen treatment has been correlated with increased FOXM1 expression [21].

We investigated the relationship between FOXM1 and ERα in breast cancer by mapping global FOXM1 binding in an ERα-positive cell line (MCF7) and an ERα-negative cell line (MDA-MB-231) using chromatin immunoprecipitation followed by high-throughput sequencing (ChIP-seq). We show that there are cell-line dependent patterns of FOXM1 binding. We identify a common set of FOXM1 binding sites in the promoter regions of cell cycle-regulating genes but additionally in MCF7 cells; the majority of binding is located in intronic and intragenic regions with a high concordance to ERα binding, similar to the distribution of FOXA1 [25], another forkhead factor. These data suggest a distinct role for FOXM1 in different cellular contexts.

## Results

### FOXM1 binding overlaps with ER binding genome-wide

**Figure 1:**
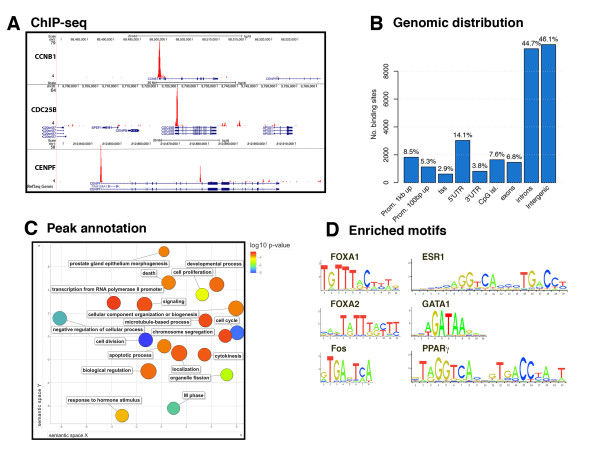
**Genome-wide distribution of FOXM1 binding in MCF7 cells**. **(a) **Three representative regions of FOXM1 binding in the promoter regions of known FOXM1 target genes; *CCNB1, CDC25B *and *CENPF*. **(b) **Global distribution of FOXM1 binding events in MCF7 cells. **(b) **REViGO analysis of the functional annotation of FOXM1 binding sites with colors representing *P*-value. **(d) **Significantly enriched motifs in the 200 bp regions around peak center.

As FOXM1 has been implicated as an important transcription factor in breast cancer, we mapped FOXM1 binding genome-wide using ChIP-seq in asynchronous MCF7 cells to determine the regulatory regions bound by FOXM1. Four biological replicates were conducted in MCF7 cells, resulting in 21,029 FOXM1 discrete binding events detected in at least two replicates. FOXM1 is known to be a key regulator of the cell cycle by regulating the transcription of genes required for G1/S and G2/M phase transition [3], and indeed we find binding in the promoter regions of many cell cycle regulating genes (Figure 1a). However, analysis of the genomic distribution of FOXM1 binding events (Figure 1b) reveals that the majority of binding is found in intronic and intergenic regions, showing a similar pattern to that of nuclear hormone receptors [26]. We carried out functional analysis of the genes within 50 kb of a binding peak and identified 34 over-represented categories with a false discovery rate (FDR) < 0.01 (Figure 1c; Table S1 in Additional file 1). As expected, the top enriched biological processes were cell proliferation, M phase regulation and mitosis. Response to steroid hormone stimulus was another enriched category. Motif enrichment analysis was conducted on the 200 bp region around the peak center of the binding peaks using the motif-based sequence analysis tool AME [27]. Amongst the top significantly enriched motifs present in regions of FOXM1 binding were members of the Forkhead, leucine zipper, GATA and nuclear hormone receptor families (examples shown in Figure 1d; additional motifs identified are shown in Table S2 in Additional file 1).

**Figure 2:**
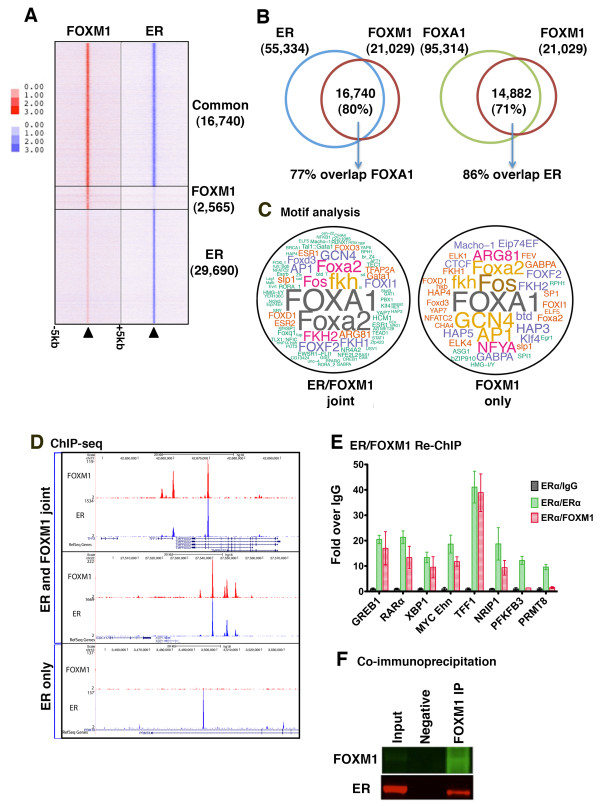
**FOXM1 shows co-operative binding with ER in MCF7 cells**. **(a) **Heat map showing overlap between FOXM1 and ER binding events. The window represents ± 5 kb regions centered on FOXM1 binding events. **(b) **Overlap of binding sites between FOXM1, ER and FOXA1 in MCF7 cells. **(c) **Motif analysis shown as a wordcloud schematic of the significantly enriched motifs present in regions bound either by both ER and FOXM1 or by FOXM1 only. The text size is inversely proportional to the log *P*-value. **(d) **Examples of genomic locations showing regions of joint FOXM1/ER binding and also ER only (PRMT8). **(e) **Re-ChIP showing co-occupancy of ER and FOXM1 in regions where both factors bind compared with regions where only ER binds (PFKFB3 and PRMT8). **(f) **Co-immunoprecipitation showing pull-down of ER with FOXM1 antibody.

As the ER binding motif was significantly enriched in the FOXM1 binding data, we overlapped the FOXM1 peaks with a previously published dataset for ERα binding [28] in asynchronous MCF7 cells. Considering overlapping peaks as those that share at least one base pair, we find a high degree of overlap between FOXM1 and ERα sites, with approximately 80% of FOXM1 binding occurring at ERα binding regions (Figure 2a, b). As FOXA1 has been shown to act as an ERα pioneer factor and is known to be required for ERα chromatin binding, we overlapped FOXM1 binding data with published genome-wide FOXA1 binding data [25]. FOXM1 and FOXA1 showed a high degree of concordance, with approximately 71% of FOXM1 binding events overlapping with FOXA1 binding events (Figure 2b).

We looked at the enriched motifs present in the regions of joint FOXM1/ERα compared to sites where only FOXM1 is located (Figure 2c). There is a high degree of overlap in the motifs identified (Table S2 in Additional file 1); in both cases the forkhead motif (FOXA1) is the most significantly enriched motif and also the leucine zipper motifs for FOS and AP1, which have previously been associated with ER and FOXA1 binding sites [29] and recently with potential FOXM1 binding regions [30]. The FOXM1-only regions did not contain ESR1 motifs or indeed any motifs for nuclear hormone receptor family members present in the joint binding regions. We identified a number of motifs present only in the regions of FOXM1 binding (Table S3 and Figure S1A in Additional file 1); interestingly, these included the NFY CCAAT motif, which has recently been associated with FOXM1 binding in promoter regions of cell cycle-regulated genes in both U2OS [31] and HeLa cells [30]. The CTCF motif was enriched in FOXM1 only regions. CTCF has been linked with both ER and FOXA1 binding sites and a recent ChIP-seq study [32] demonstrated a set of co-localized CTCF/ER or CTCF/FOXA1 binding sites in MCF7 cells; however, the majority were CTCF/FOXA1 joint sites, with the CTCF/ER sites located predominantly in promoter regions.

To confirm that FOXM1 and ERα can simultaneously co-occupy the same chromatin location, re-ChIP experiments followed by quantitative PCR (qPCR) were performed focusing on regions of co-binding and, as controls, regions with only ERα binding (example regions are shown in Figure 2d). ERα ChIP was performed first using asynchronous MCF7 cells followed by FOXM1 ChIP or IgG as a negative control. Results confirmed (Figure 2e) that these transcription factors simultaneously co-occupy the same genomic locations. Using co-immunoprecipitation Figure 2f), we observed a direct interaction between FOXM1 and ERα, suggesting that FOXM1 binds as part of the ER transcriptional complex [33].

### FOXM1 binding at co-bound sites is dependent on ERα

**Figure 3:**
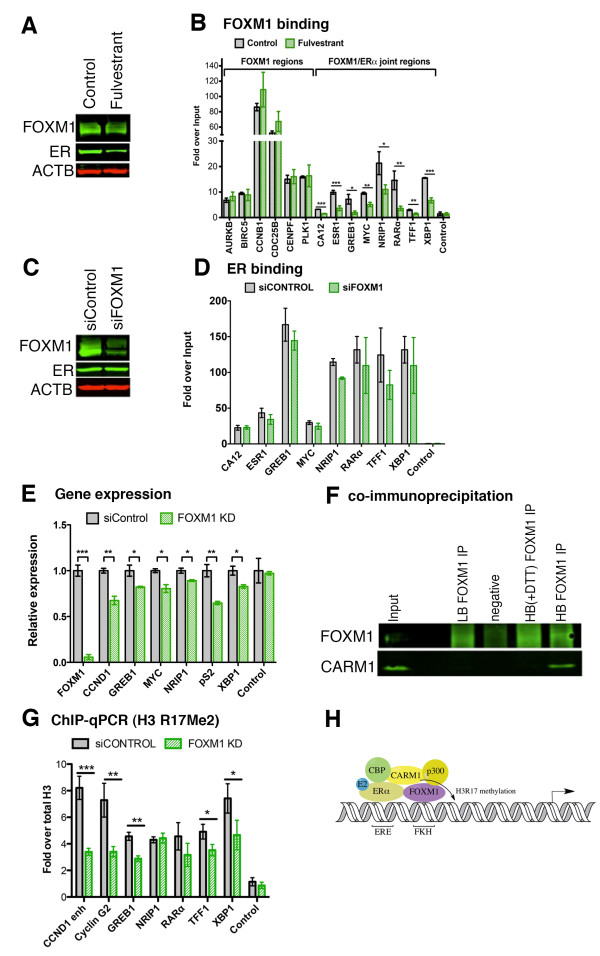
**FOXM1 binding at ERα co-bound sites is dependent on ERα**. **(a) **FOXM1 binding was assessed following depletion of ER by fulvestrant (10 nM) treatment for 3 h. Western blot showing depletion of ERα whilst FOXM1 protein levels are unchanged. **(b) **ChIP for FOXM1 was followed by qPCR to assess binding at ERα co-bound sites and FOXM1-only binding sites. ERα binding was assessed following depletion of FOXM1 by siRNA treatment for 48 h. **(c) **Western blot showing depletion of FOXM1 whilst ERα protein levels are not significantly changed. **(d) **ChIP for ER followed by qPCR to detect binding at FOXM1 co-bound regions. Depletion of FOXM1 affects ERα-regulated gene expression. **(e) **qPCR of ERα-regulated gene expression in MCF7 cells treated with siRNA for siControl or FOXM1 for 48 h. Nascent and total mRNA levels were measured. FOXM1 interacts directly with the co-activator CARM1 and regulates CARM1-mediated histone H3 arginine methylation. **(f) **Co-immunoprecipitation showing pull-down of CARM1 with FOXM1 antibody using either low binding (LB) or high binding (HB) immunoprecipitation buffers additionally supplemented with dithiothreitol (DTT). **(g) **ChIP for methylated arginine 17 on histone H3 followed by qPCR for regions of FOXM1 and ER co-binding. Data are normalized to total H3 in MCF7 transfected with siRNA for 48 h (siControl or FOXM1). **(h) **Proposed simplified model for FOXM1/ER/CARM1 complex in transcription regulation at enhancer regions. Data representative of triplicate experiments ± standard deviation. *P < 0.05, **P < 0.01, ***P < 0.001.

To investigate the relationship between FOXM1 and ERα at sites of co-binding, we looked at the recruitment of FOXM1 following treatment of MCF7 cells with fulvestrant, an estrogen receptor antagonist [34] that forms an unstable complex with ER, thereby increasing the rate of degradation. Following a 3 hour treatment with fulvestrant, there was a significant reduction in ERα protein but FOXM1 protein and transcript levels were unchanged (Figure 3a; Figure S2 in Additional file 1). We performed ChIP followed by qPCR for regions where FOXM1 and ERα both bind (determined by analysis of ChIP-seq data; Figure 2d) and regions where only FOXM1 binds. This showed (Figure 3b) a significant reduction in FOXM1 binding (*P *< 0.05) at regions where ERα also binds but not at ERα-negative regions such as the promoter of *PLK1*. We also looked at the mRNA levels of the target genes of the binding regions (Figure S2 in Additional file 1) and observed a significant reduction in expression for genes that are co-bound by FOXM1 and ERα (*P *< 0.05). This result suggests that ERα binding is required for FOXM1 recruitment at co-bound regions and confirms the re-ChIP results showing co-occupancy of FOXM1 and ERα at these same genomic loci. In contrast, treatment with the proteasome inhibitor MG132, which is known to reduce FOXM1 expression [35], reduces FOXM1 binding at both ERα co-bound and also ERα-negative regions (Figure S3 in Additional file 1). We assessed the effect of small interfering RNA (siRNA)-mediated knockdown of FOXM1 on ERα binding by ChIP-qPCR. The level of ERα protein was not significantly altered by FOXM1 knockdown as shown by western blot (Figure 3c) and we did not see any significant differences in ERα binding at known binding sites (Figure 3d), suggesting that FOXM1, unlike FOXA1, is not necessary for ERα to bind to the chromatin. Previous studies have shown that FOXM1 expression is required for ERα-induced proliferation [21] and indeed we observed that depletion of FOXM1 significantly reduced the expression of a number of ERα-regulated genes (Figure 3e), suggesting a functional role for FOXM1 at these co-bound sites.

### Interaction of FOXM1 with ERα and CARM1 co-activator

Since FOXM1 was not required for ERα to bind to DNA, we hypothesized that FOXM1 may be involved in tethering co-factors to the ERα complex. We used an initial proteomic screen (data not shown) to identify FOXM1 interacting proteins and checked for proteins known to be part of the ERα transcriptional complex [36]. Using this method we found a possible interaction with co-activator-associated arginine methyltransferase (CARM1), one of the key co-factors involved in ERα signaling [37,38]. We demonstrate (Figure 3f) using co-immunoprecipitation that there is a direct interaction between these proteins and propose that this might be important for regulating transcriptional activity at these sites. CARM1 is known to act as a transcriptional regulator by methylation of arginine residues in histones and co-activator proteins such as p160 and CBP/p300 [39]. We therefore investigated whether FOXM1 binding at ERα co-bound sites regulated the methylation activity of CARM1. ChIP-qPCR for H3 arginine 17 modifications in MCF7 cells treated with siRNA for FOXM1 showed a significant decrease following depletion of FOXM1 protein at the sites of co-binding (Figure 3g). This suggests that FOXM1 may assist in recruitment of co-factors, such as CARM1, to ERα binding sites (Figure 3h).

### FOXM1 binding is altered in ERα-negative cell lines

**Figure 4:**
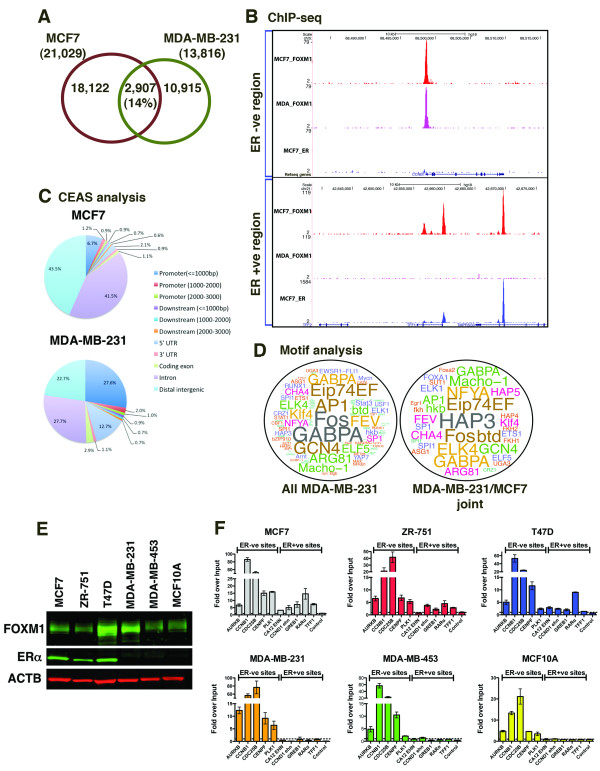
**FOXM1 shows differential binding according to ERα status in breast cancer cells**. **(a) **Overlap of FOXM1 binding events between MCF7 (ER-positive cells) and MDA-MB-231 (ER-negative cells). **(b) **Examples of genomic regions showing FOXM1 binding in MCF7 and MDA-MB-231 cells compared to ER binding in MCF7 cells, contrasting ER-negative regions to ER-positive regions. **(c) **CEAS analysis showing genomic distribution of FOXM1 binding events in MCF7 and MDA-MB-231. **(d) **Motif analysis shown as a wordcloud schematic of the significantly enriched motifs present in FOXM1 bound regions in MDA-MB-231 cells and in binding regions overlapping in MCF7 cells. The text size is inversely proportional to the log *P*-value. **(e) **Western blot showing FOXM1 and ERα protein levels in six breast cancer lines. **(f) **FOXM1 ChIP followed by qPCR was performed in ER-positive breast cancer cell lines (MCF7, T47D and ZR-751), ER-negative breast cancer cell lines (MDA-MB-231 and MDA-MB-453) and the normal breast cell line MCF10A to compare FOXM1 binding at known regions of co-binding with ER (from MCF7 ChIP-seq data) to ER-negative binding regions. Data representative of triplicate experiments ± standard deviation.

To explore the hypothesis that FOXM1 is recruited to DNA by ERα in ER-positive breast cancer cells, we performed ChIP-seq to map FOXM1 binding in the ERα-negative cell line MDA-MB-231 and compared the binding events to ERα-positive MCF7 (Figure 4a). We observed little overlap in FOXM1 binding events between the two cell lines, with only 14% of MCF7 FOXM1 binding occurring at the same genomic location in MDA-MB-231 cells. Approximately 52% of the common binding regions in the two cell lines were found at sites where ERα is not bound in MCF7 cells (example shown in Figure 4b), implying a subset of biologically reproducible ERα-independent FOXM1 binding events. FOXM1 binding in the different cell lines showed different genomic distributions (Figure 4c) with a significant enrichment of binding in promoter regions in the MDA-MB-231 cells when compared to MCF7 cells (approximately 31% compared to approximately 9% in MCF7, compared to approximately 2% for the genome average distribution) and 5' UTR (approximately 13% compared to approximately 2% in MCF7 and approximately 0.4% for the genome average distribution) whilst binding in the intergenic and intronic regions was reduced (approximately 50% compared to approximately 85% in MCF7 and 92% for the genome average distribution). Most of the shared FOXM1 binding events are in promoter regions (Figure S4 in Additional file 1).

Motif analysis of MDA-MB-231 binding peaks showed little overlap with those identified in MCF7 binding peaks in regions of joint binding with ERα; however, comparison with the top enriched motifs in the FOXM1 binding regions in MCF7 cells revealed a similar pattern of cis-elements present (Figure 4d; Table S4 in Additional file 1). Interestingly, although present, the FKH motif was not amongst the top 50 enriched motifs in this cell line. The most significantly enriched motifs were for members of the Ets and leucine zipper families of transcription factors; also present was the NFY CCAAT-binding motifs, which were recently shown to be present in sites of FOXM1 binding in U2OS cells [31]. This suggests that while there is a core set of common FOXM1 binding sites, there are also cell line-specific binding patterns. The lower enrichment of FKH motifs in the MDA-MB-231 cells suggests recruitment by other transcription factors. We found enrichment of the helix-loop-helix transcription factor family motif in the MDA-MB-231 cell line and have observed a high level of concordance with c-MYC binding in this cell line (data not shown).

ChIP-qPCR of FOXM1 binding sites was carried out in additional ERα-positive (namely ZR-751 and T47D) and -negative (namely MDA-MB-453 and MCF10A) cell lines (Figure 4e, f) looking at regions identified in MCF7 cells as FOXM1-only sites or sites of ERα co-binding. We found that FOXM1 binding in all cell lines tested was enriched in the promoter regions of cell cycle-regulating proteins whilst in the ERα-positive cells (ZR-751 and T47D) binding was also detected in regions known to be ERα binding sites in MCF7 (GREB1, RARα and TFF1) but not in the ERα-negative cells (MDA-MB-453 and MCF10A). The pattern of FOXM1 binding was confirmed using two additional antibodies with specificity to different regions of FOXM1 (Figure S5 in Additional file 1) in MCF7 cells.

### Inhibition of FOXM1 binding in MCF7 cells modulates binding at specific sites

**Figure 5:**
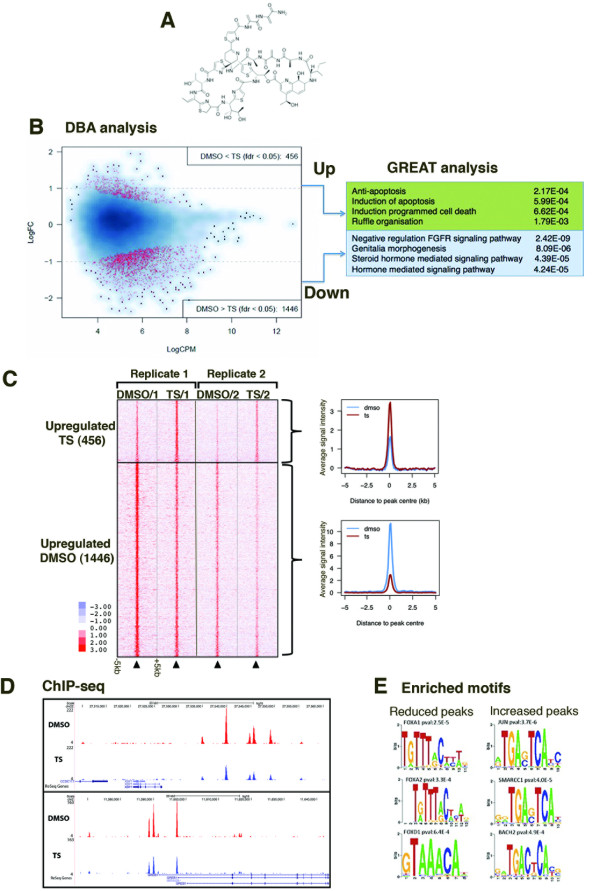
**Treatment of MCF7 cells with thiostrepton alters FOXM1 binding genome-wide**. **(a) **Molecular structure of the thiazole antibiotic thiostrepton. **(b) **Differential binding analysis (DBA) was used to identify significantly (FDR < 0.05) differentially bound peaks in DMSO compared to thiostrepton (TS)-treated cells from four replicates. The dots shown in red represent peaks where FOXM1 binding is significantly increased/decreased compared to the control. Enrichment of Gene Ontology processes associated with the differentially bound peaks was determined using GREAT; the top four significantly enriched processes in the upbound and downbound regions are shown. **(c) **Heatmap of DBA binding events for two of the replicates showing FOXM1 binding signal intensity in regions up- or down-regulated by thiostrepton treatment in a window of ± 5 kb and total signal intensity of differentially bound peaks showing fold change in binding in thiostrepton-treated compared to control cells. **(d) **Examples of genomic loci where FOXM1 binding is reduced following treatment of MCF7 cells with thiostrepton. **(e) **Motif analysis of the 200 bp region around peak center in the differentially bound peaks.

To study the effect of inhibition of FOXM1 binding on gene expression and cellular phenotype, we treated MCF7 cells with the thiazole antibiotic thiostrepton (Figure 5a). This molecule inhibits FOXM1 activity [40,41] and we have previously shown that thiostrepton interacts directly with FOXM1 inhibiting DNA binding [42] at certain genomic loci. In this study we aimed to extend this analysis genome-wide. Thiostrepton is known to reduce FOXM1 expression and as such we treated cells for 4 hours, a timepoint where there is no significant change in FOXM1 protein level [42] and performed ChIP-seq to map global changes in FOXM1 binding. ChIP-seq was performed in quadruplicate with at least 25 million sequencing reads per replicate (Table S5 in Additional file 1). FOXM1 binding events were identified using MACS [43] and any FOXM1 binding events present in at least two out of the four replicates were considered. Differential binding analysis (DBA; see Materials and methods) was used on this dataset of 21,494 FOXM1 binding events to identify binding events that were statistically differentially bound in either the control or thiostrepton-treated condition. Using an FDR < 0.05, a total of 1,902 differentially bound peaks were identified from the replicate sets of control/thiostrepton-treated samples (Figure 5b; Additional file 2), with the majority of FOXM1 binding events showing decreased binding after treatment with thiostrepton (1,446, representing approximately 76% of all differential FOXM1 binding events) (Figure 5c), consistent with our previous report that thiostrepton inhibits FOXM1 DNA binding [42]. Examples of differentially bound regions are shown in Figure 5d. Pathway analysis using GREAT (Genomic Region Enrichment Analysis Tool) [44] on regions associated with differentially bound peaks (Figure 5b) showed that regions with increased FOXM1 binding after thiostrepton treatment were enriched for apoptosis signaling pathways, consistent with the known induction of apoptosis with thiostrepton [45]. Interestingly, in the FOXM1 binding regions with decreased binding after thiostrepton treatment, the top enriched processes were related to the steroid hormone-signaling pathway as well as fibroblast growth factor receptor (FGFR) signaling, a key pathway involved in epithelial to mesenchymal transition and cell proliferation [46,47]. This supports the qPCR data (Figure 3e) showing decreased expression of ERα-regulated genes following depletion of FOXM1, suggesting a functional role for FOXM1 in ERα activity at co-binding sites. Cis-regulatory element annotation system (CEAS) analysis of the DBA peaks (Figure S6 in Additional file 1) showed that the increased binding was enriched in promoter regions. Interestingly, motif analysis of the DBA peak regions (Figure 5e; Table S6 in Additional file 1) showed that three out of the four enriched motifs were for FKH factors in the down-regulated regions whilst there was no enrichment of FKH motifs in the up-regulated regions.

**Figure 6:**
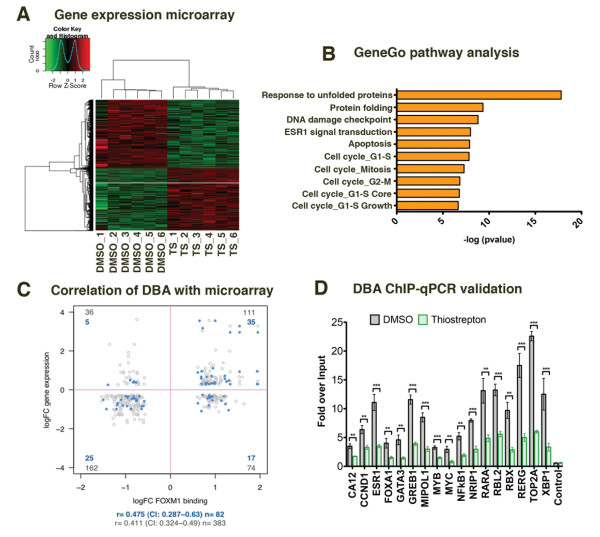
**Changes in FOXM1 binding induced by thiostrepton correlate with an altered gene expression profile**. **(a) **Microarray analysis of gene expression changes following treatment of MCF7 cells with DMSO or thiostrepton (TS) for 6 h. Only genes with an FDR < 0.01 were considered, resulting in 5,638 differentially expressed genes. Heat map shows differentially expressed genes for the six replicates. **(b) **GeneGo analysis of the differentially expressed genes showing the top ten enriched biological processes. **(c) **Correlation between differentially bound FOXM1 peaks from DBA analysis and differentially expressed genes from the microarray analysis following thiostrepton treatment. Blue dots represent peaks within 10 kb of the gene TSS, grey dots < 50 kb from the TSS. **(d) **FOXM1 ChIP was performed on DMSO or thiostrepton-treated MCF7 cells and qPCR was used to check FOXM1 binding in regions identified in DBA analysis as significantly differentially bound. Data representative of triplicate experiments ± standard deviation. *P < 0.05, **P < 0.01, ***P < 0.001.

To test whether the differentially bound FOXM1 regions affect gene expression, we performed gene expression analysis on MCF7 cells after treatment with dimethylsulphoxide (DMSO) or thiostrepton for 6 hours. Using an FDR < 0.01 and log ratio ≥ 0.5, there were 2,322 differentially expressed genes (Figure 6a), with 1,098 down-regulated and 1,224 up-regulated genes. Gene Ontology analysis of the function annotations for the differentially expressed genes showed that out of the top ten categories based on *P*-value, cell cycle regulation and apoptosis pathways were over represented (Table S7 in Additional file 1 lists the top 10 up/down-regulated genes and Additional file 3 lists all differentially expressed genes). Interestingly the ERα signaling pathway was also highly enriched, again supporting the importance of FOXM1 as a key factor in ERα signaling.

We integrated the differentially expressed genes and differentially bound peaks (identified from the DBA) using either a 10 or 50 kb window. For genes within 10 kb of the nearest peak there was a correlation of 0.48 (95% confidence intervals 0.29 to 0.63) between the thiostrepton regulated FOXM1 binding events and the thiostrepton regulated genes (Figure 6c). Extending the window to 50 kb resulted in a correlation of 0.41 (95% confidence intervals 0.32 to 0.49). Using the 50 kb window we identified 383 peaks associated with genes (Additional file 4). To validate these results we selected 17 down-regulated and 5 up-regulated genes (Table S8 in Additional file 1) and performed independent ChIP experiments with MCF7 cells treated with DMSO/thiostrepton for 4 hours followed by qPCR for the peak region from the DBA (Figure 6d). We found that there was a significant reduction (*P *< 0.05) in binding in all of the down-regulated regions corresponding with the reduced expression in the microarray dataset. However, for the up-regulated regions we found that the level of enrichment was too low to verify reproducibly.

### Inhibition of FOXM1 regulates a gene-signature correlated with prognosis

Gene Set Enrichment Analysis (GSEA) [48] was used to compare thiostrepton-regulated genes containing a FOXM1 binding peak (FDR < 0.05) within 50 kb of the transcription start site (TSS) (405 unique genes) with curated gene sets in the Molecular Signature Database (MSigDB). We analyzed the up- and down-regulated genes as separate lists. Using the down-regulated gene set (198 identified genes) the top 10 significantly overlapping sets were all related to breast cancer and ERα signaling (Table S9 and Figure S7 in Additional file 1). There was a significant correlation between the thiostrepton down-regulated gene set and genes shown in previous studies to be estrogen-regulated in both ERα-positive cell lines [49,50] and tumor samples [51,52] and gene sets related to resistance to endocrine therapy [53-55]. Similar analysis using the genes up-regulated by thiostrepton (111 identified genes) revealed that the ten most significantly overlapped gene sets were from diverse studies (Table S10 in Additional file 1), none of which related to breast cancer or ERα signaling.

**Figure 7:**
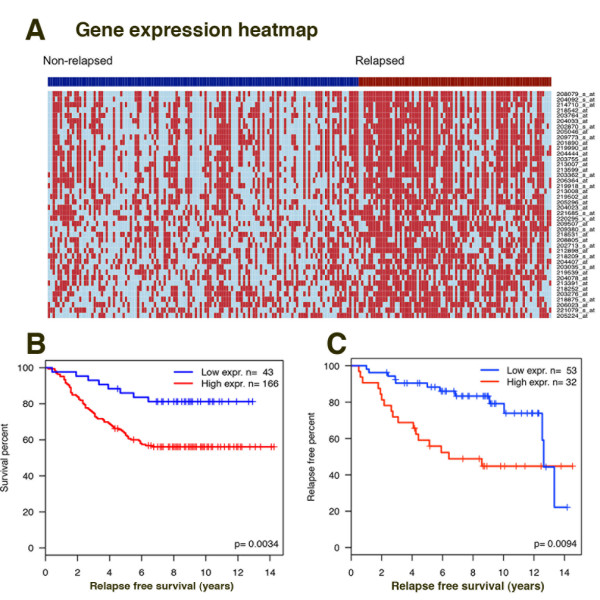
**FOXM1 regulates genes associated with poor prognosis in ER-positive breast cancer patients**. Comparison of the thiostrepton down-regulated genes with a FOXM1 binding site (± 50 kb TSS) with expression data from a breast cancer patient array dataset containing 286 samples (209 ER positive) with patient survival outcome [56] showed a set of 42 probes (38 unique genes) significantly associated with poor prognosis. **(a) **Heat map showing gene expression in the ER-positive patients. Patients are grouped into good prognosis (non-relapsed, blue ribbon) and poor prognosis (relapsed, red ribbon). Gene expression is represented as light blue or red for patients with expression below or above the gene median, respectively. **(b) **Kaplan-Meier plot showed a significant positive correlation between patient relapse and expression of genes down-regulated by thiostrepton and associated with FOXM1 binding sites. **(c) **Kaplan-Meier plot using the same gene signature set as in (b) in an independent breast cancer dataset [57] confirmed correlation with patient survival.

As FOXM1 itself is a poor prognosis marker in ERα breast cancer (Figure S8 in Additional file 1) we tested whether the genes regulated by thiostrepton and with a FOXM1 binding site within 50 kb of the TSS were correlated with clinical outcome in breast cancer. In this case we again considered the up- and down-regulated genes separately and tested whether there was any correlation with patient survival using a publically available data set of 286 breast cancer patients with associated survival data [56]. We restricted analysis to ERα-positive patients (209) and used this set to test for enrichment of genes correlated with prognosis in the thiostrepton-regulated gene sets. In the case of the up-regulated genes there was no evidence of overrepresentation (*P *= 0.217). However, for the down-regulated genes there was a significant correlation (*P *< 10^-6^) with 38 genes identified (Table S11 in Additional file 1) whose expression is related to prognosis (high expression correlated to reduced time to relapse; Figure 7a, b; Figure S9 in Additional file 1). Survival analysis using this 38 gene-signature showed a significant correlation (*P *< 0.0034) with patient survival (Figure 7b), with high expression correlating with reduced time to relapse; these results were also confirmed using another independent dataset (*P *= 0.0094) [57] (Figure 7c; Figure S10 in Additional file 1). We looked for known functional interactions within the genes identified using STRING (Search Tool for the Retrieval of Interacting Genes/Proteins) [58] and found high confidence interactions linking 20 proteins relating to mitotic spindle formation, chromosome segregation and cell cycle regulation (Figure S11 in Additional file 1).

This confirms the therapeutic potential of targeting FOXM1, as thiostrepton treatment reduces the expression of genes whose high expression correlates with poor patient outcome in breast cancer.

## Discussion

The forkhead factor FOXM1 was first linked with cancer in 2002 [59] and has subsequently been shown to be involved in cancer initiation [13], progression [60] and prognosis [10] in diverse tissue types. FOXM1 is a known regulator of cell proliferation and mitotic spindle assembly genes but has also been linked with regulating epithelial to mesenchymal transition, angiogenesis, metastasis and more recently with induction of DNA methylation [61].

In this study we focus on the role of FOXM1 in breast cancer with the aim of mapping global FOXM1 binding sites in both ER-positive and -negative cancer cell lines and relating this to biological function. We show that FOXM1 binding occurs in the promoter regions of many cell cycle-related genes and also genes known to regulate mitotic spindle assembly; *BUB1, ASPM, MAD2L1 *and members of the kinesin family. We also confirmed binding in target genes such as *CAV1 *[62] and MMP family members; *MMP2 *and *8, VEGF *and *FGF *linking with the role of FOXM1 in metastasis.

The high degree of overlap between FOXM1 binding and ERα binding in MCF7 is of significant interest as previous studies have shown co-regulation of these factors [21,22] and importantly that FOXM1 expression is essential for ERα-induced proliferation. We show that FOXM1 and ERα bind to the chromatin at the same place at the same time; however, the presence of FOXM1 at ERα co-bound chromatin sites is not required for ERα recruitment but is necessary for the loading of other cofactors to the ER transcriptional complex, such as CARM1. CARM1 is known to activate transcription by methylating histone H3 arginine residues [63] and constitutes a key part of nuclear hormone receptor transcriptional complexes acting in conjunction with other coactivators, such as CBP/p300, SRC-2 and GRIP1 [33,64]. FOXM1 has previously been shown to interact directly with CARM1 binding factors such as p300/CBP [65] but, to our knowledge, this is the first time that a direct interaction has been shown between FOXM1 and CARM1.

The differences in FOXM1 binding with ERα status in breast cancer cells contribute another dimension to the already complex mechanisms known to regulate FOXM1 transactivation. FOXM1 activity is regulated by multiple protein-protein interactions; during the cell cycle the temporal control required for precise G2/M transition is regulated both by phosphorylation steps involving different Cyclin/Cdks [66-68] and by inhibition by B55a, a subunit of protein phosphatase 2A [69]. Transcriptional activity is modulated by recruitment of coactivators and interaction with other transcription factors [70]. The data from our study suggest another mode of interaction of FOXM1, in this case co-binding with ERα in ERα-positive breast cancer cells.

A similar role for FOXM1 in transcriptional regulation by recruitment of co-factors at specific genomic locations was proposed in a recent study by Carr *et al. *[71]. They showed that FOXM1 represses GATA-3 expression in the mammary gland by recruiting the methyltransferase DNMT3b to binding sites within the GATA-3 promoter, thereby leading to methylation-induced gene silencing.

Inhibition of FOXM1 binding in MCF7 cells using thiostrepton confirmed the importance of this transcription factor in the regulation of ERα signaling pathways. Analysis of the regions where binding was significantly reduced showed that these were *cis*-regulatory regions involved in ERα pathways and indeed the gene expression changes confirmed that ERα signaling pathways were affected. Somewhat surprisingly, FOXM1 binding was increased at a number of genomic sites following thiostrepton treatment; however, these sites did not contain consensus FKH binding motifs and may represent a redistribution of FOXM1 in a similar manner to that described for the androgen receptor following FOXA1 depletion [72]. In the absence of FOXA1, androgen receptor binding was delocalized around the genome, generating, in addition to FOXA1 dependent-sites, both a subset of FOXA1-inhibited sites and a subset of FOXA1-independent binding sites where androgen receptor recruitment may occur via the presence of transcription factors other than FOXA1 with pioneer activity. Furthermore, a recent publication showed that FOXM1 has an atypical DNA binding mechanism [31], with binding at some genomic sites occurring via recruitment and binding to the MMB transcriptional activator complex. This mechanism not only explains how binding is increased at certain sites after inhibition of direct DNA binding but also fits with the binding pattern we observed in MDA-MB-231 cells compared to MCF7 cells, where the FKH motif was poorly enriched compared to other motifs such as the ETS family members. In fact we cannot rule out that some of the enriched FKH consensus sites in MCF7 cells actually represent FOXA1 binding motifs with FOXM1 tethered via other transcriptional co-factors. Thus, FOXM1, as a key regulator of the cell cycle, may show cell line-dependent patterns of DNA binding due to recruitment by different transcriptional complexes responsible for driving the proliferation in a particular cellular context.

As FOXM1 expression has previously been correlated with prognosis in breast cancer we used our dataset to identify a novel FOXM1-regulated gene set significantly correlated with ER-positive breast cancer prognosis and drug resistance, thus confirming previous studies showing that FOXM1 over-expression is associated with resistance to drug treatment [19,21]. This gene-signature of 38 FOXM1-regulated genes down-regulated by thiostrepton treatment was predictive of prognosis in ER-positive breast cancer patient datasets. Within this set are many well described FOXM1 target genes, such as those involved in mitotic spindle formation; however, there are a number of interesting novel FOXM1 target genes, such as *ABCC5*, a transporter protein associated with multi-drug resistance that may provide useful insights for future studies into the role of FOXM1 in breast cancer.

## Conclusions

We have demonstrated that FOXM1 shows distinct patterns of binding depending on ERα status in breast cancer cells, but within an ER-positive context FOXM1 plays an important role in ERα signaling pathways. Specifically, FOXM1 regulates a gene signature that correlates with poor prognosis in breast cancer patients, supporting the therapeutic potential in targeting FOXM1 in ER-positive breast cancer.

## Materials and methods

### Cell culture

Human MCF7, MDA-MB-231, ZR-751, T47D, MDA-MB-453 and MCF10A cell lines were obtained from the ECACC (European Collection of Animal Cell Cultures) and grown in DMEM or RPMI (ZR-751, T47D) supplemented with 10% FBS and the MCF10A in MEGB (Lonza Biologics, Slough, Berkshire, UK). Thiostrepton (Sigma-Aldrich Company Ltd, Gillingham, Dorset, UK) and MG132 (Sigma) were made up as stock solutions of 10 mM in DMSO and used at a final concentration of 10 μM (thiostrepton) and 3 μM (MG132) and fulvestrant (ICI 182780) was added at a final concentration of 10 nM.

### Western blots

Western blots were performed using antibodies for anti-FOXM1 (sc-502) and anti-ER (sc-543) from Santa Cruz Biotechnology (Dallas, Texas, USA) and anti-β-actin (ab6276) from Abcam (Cambridge, Cambridgeshire, UK). Further details are provided in the Supplementary materials and methods in Additional file 1.

### Quantitative real-time PCR analysis

MCF7 cells were treated with thiostrepton/MG132/fulvestrant and RNA was collected after the indicated timepoints and qPCR was performed using Power Sybr mix (ABI, Warrington, Cheshire, UK) on a CFX96 Real-time thermal cycler (Bio-Rad, Hemel Hempstead, Hertfordshire, UK). Further details and primer sequences are given in Additional file 1.

### Small interfering siRNA

MCF7 cells were transfected with Dharmacon siGenome pools for FOXM1 or siCONTROL at 15 nM using lipofectamine 2000. Knockdown of FOXM1 protein levels was confirmed by western blotting after 48 h incubation.

### Microarray analysis

MCF7 cells were treated with either thiostrepton or DMSO for 6 h. Each treatment was carried out in replicates of six. Gene expression analysis was carried out on Illumina Human HT12 version 4 bead arrays. Details of analysis are given in the Supplementary materials and methods in Additional file 1. Data are available through the NCBI's Gene Expression Omnibus [73] using GEO Series accession number GSE40767.

### Chromatin immunoprecipitation

ChIP experiments were performed as previously described [74]. Antibodies used were anti-FOXM1 (Santa Cruz sc-502, Genetex GTX1000276, Genetex GTX102170 [GeneTex, Irvine, California, USA]) and anti-ER (Santa Cruz sc-543), anti-histone H3 (Abcam ab1791) and anti-histone H3 (asym-dimethyl Arg17; Novus NB21-1132 (Novus Biologicals, Cambridge, Cambridgeshire, UK)). Experimental details and primer sequences are available in Additional file 1.

### ChIP-sequencing experiments

To detect FOXM1 binding sites affected by thiostrepton versus DMSO, ChIP-Seq experiments were performed in four biological replicates in MCF7 cells and in two biological replicates in MDA-MB-231 (Table S5 in Additional file 1). ChIP DNA was processed for Illumina sequencing as previously described [74]. Details of analysis are in Additional file 3. Data are available through the NCBI's Gene Expression Omnibus [73] using GEO Series accession number GSE40767.

### Analysis of differential binding

To identify regions of differential FOXM1 binding between the control (DMSO) and thiostrepton-treated samples, a general linear model was fitted to each putative binding site to test for the difference in read count between treatments (see Supplementary materials and methods in Additional file 1 for detection and definition of binding sites). Model fitting and testing was performed using the Bioconductor library edgeR [75] using the function estimateGLMTagwiseDisp for estimating the dispersion parameter of the negative binomial distribution and glmFit and glmLRT for fitting and testing the difference of treatment of each binding site [76]. The heatmaps in Figures 2 and 5 were prepared with Java Treeview [77].

### Motif analysis and genomic distribution of binding events

The CEAS [78] function in Cistrome [79] was used to functionally annotate binding sites. Known transcription factor motifs significantly enriched in the binding sites were identified with AME [27]. The -log *p*-value of significance was used to scale the word clouds using the R package wordcloud [80].

### Gene Ontology pathway analysis

Gene Otology pathway enrichment was performed using GeneGo metacore (MetaCore from Thomson Reuters (New York, USA)) and visualized with REViGO (Reduce and Visualize Gene Ontology) [81].

### Re-ChIP

Re-ChIP was performed as described [82] using the same antibodies as for ChIP and normal rabbit IgG (2729) from Cell Signaling Technology (Danvers, Massachusetts, USA).

### Co-immunoprecipitation

Experiments were performed using the nuclear co-immunoprecipitation kit from Active motif (Carlsbad, California, USA) with pull-down using FOXM1 (Santa Cruz sc-502) following the manufacturer's protocol with immunoprecipitation carried out using either the low or high buffers provided supplemented with 1X protease inhibitor cocktail and 1 mM dithiothreitol (DTT). Detection was performed by western blotting using ERα (Novacastra, Milton Keynes, Buckingham, UK) and CARM1 (Santa Cruz sc-5421) with LiCor IRDye secondary antibodies; 800LT goat anti-mouse, 680LT goat anti-rabbit and 680LT donkey anti-goat. Further details are in Supplementary materials and methods in Additional file 1.

### Survival analysis

The clinical and gene expression dataset GSE2034 [56] was analyzed to investigate whether genes regulated by thiostrepton are involved in poor cancer prognosis; only the 209 ER-positive samples were considered. Details of the analysis are in Supplementary materials and methods in Additional file 1). Gene Set Enrichment Analysis (GSEA) [48] was performed using the web-based tool [83] to compare gene lists with the Molecular Signatures Database (MSigDB).

### Statistical analysis

All statistical analyses not described above were performed with GraphPad prism software or R [84]. The tests for difference between means were performed using the two-tailed Student's *t*-test. If not otherwise stated, *P*-value < 0.05 was considered statistically significant. Error bars represent standard deviations.

## Abbreviations

bp: base pair; CEAS: cis-regulatory element annotation system; ChIP: chromatin immunoprecipitation; DBA: differential binding analysis; DMSO: dimethylsulphoxide; ER: estrogen receptor; FDR: false discovery rate; FKH: Forkhead; GREAT: Genomic Region Enrichment Analysis Tool; qPCR: quantitative polymerase chain reaction; REViGO: Reduce and Visualize Gene Ontology; siRNA: small interfering RNA; TSS: transcription start site; UTR: untranslated region.

## Competing interests

The authors declare that they have no competing interests.

## Authors' contributions

DS, CSR, SB and JSC designed all experiments. DS and CSR performed the experimental work. DB performed analysis and processing of ChIP-seq data and statistical analysis. All authors contributed to the interpretation of the data. The manuscript was written by DS, CSR and DB with help from other authors. All authors read and approved the final manuscript.

## Supplementary Material

Additional file 1 **Supplementary materials and methods, tables and figures as mentioned in the text**. Click here for file

Additional file 2 **FOXM1 binding peaks in MCF7 cells**. Excel spreadsheet containing the location of FOXM1 binding peaks identified using MACS in replicate samples of MCF7 cells treated with DMSO or thiostrepton. Differential bound peaks (FDR < 0.05) were identified using edgeR. Click here for file

Additional file 3 **Differentially expressed gene list from microarray analysis**. Excel spreadsheet with details of differentially expressed gene (FDR < 0.01) from microarray analysis for DMSO versus thiostrepton-treated MCF7 cells. Click here for file

Additional file 4 **FOXM1 binding peaks from DBA versus expression changes from microarray**. Excel spreadsheet containing LogFC values for differential expression of genes with TSSs located within ± 50 kb of differentially bound FOXM1 identified from DBA in MCF7 cells treated with DMSO or thiostrepton. Click here for file
