# Supplementary material for: Genome-wide mapping of FOXM1 binding reveals co-binding with estrogen receptor alpha in breast cancer cells
Source: Genome Biol. 2013 Jan 24;14(1):R6. doi: 10.1186/gb-2013-14-1-r6 (PMC3663086; doi:10.1186/gb-2013-14-1-r6)

Supporting Information:

Genome-wide mapping of FOXM1 binding reveals co-binding with estrogen receptor alpha in breast cancer cells.

Deborah A Sanders, Caryn S Ross-Innes, Dario Beraldi, Jason S Carroll and Shankar Balasubramanian

Table of Contents:

Supplementary Materials and methods

Supplementary References

**Table S1**	Revigo analysis of FOXM1 binding sites
**Table S2**	Motif analysis of joint FOXM1/ER binding regions
**Table S3**	Motif analysis of FOXM1 only binding regions
**Table S4**	Motif analysis of FOXM1 peaks in MDA-MB-231 vs MCF7 cells
**Table S5**	ChIP-Seq libraries used for mapping FOXM1 binding sites.
**Table S6**	Motif analysis of thiostrepton-regulated peaks
**Table S7**	Differentially expressed genes from microarray
**Table S8**	DBA regions for validation
**Table S9**	GSEA analysis of Thiostrepton downregulated genes
**Table S10**	GSEA analysis of Thiostrepton upregulated genes
**Table S11**	Thiostrepton regulated genes associated with prognosis

**Figure S1**	Motif and CEAS analysis of FOXM1 binding sites in MCF7 cells
**Figure S2**	qPCR following fulvestrant treatment of MCF7 cells
**Figure S3**	FOXM1 ChIP-qPCR of MCF7 cells treated with Fulvestrant/MG132
**Figure S4**	CEAS analysis of FOXM1 binding in MCF7 vs MDA-MB-231
**Figure S5**	FOXM1 ChIP-qPCR
**Figure S6**	CEAS analysis of FOXM1 DBA regions
**Figure S7**	GSEA analysis of Thiostrepton downregulated genes
**Figure S8**	Kaplan-Meier survival analysis
**Figure S9**	Clustering analysis with Wang breast cancer dataset
**Figure S10**	Heatmap and clustering analysis with Loi breast cancer dataset
**Figure S11**	STRING protein interactions

Supplementary Materials and methods

Western blotting

Cell lysates were prepared using RIPA buffer (20 mM Tris-HCl, pH 7.5, 150 mM NaCl, 1 mM Na_2EDTA, 1 mM EGTA, 1% NP-40, 1% sodium deoxycholate, 2.5 mM sodium pyrophosphate, 1 mM ß-glycerophosphate, 1 mM Na_3VO_4) with proteasome inhibitors (Roche). Lysate was agitated for 30 min at 4°C by end-to end rotation, supernatant collected following centrifugation (13000 rpm, 4°C, 10 min) and protein concentration measured by BCA (Pierce) assay. Samples loaded onto 4-12 % Tris-Glycine mini gels (Invitrogen), purified by SDS-PAGE and transferred to nitrocellulose membrane (Invitrogen). Membranes were incubated in Odyssey blocking buffer (LiCor) for 1 h at room temperature and probed with FOXM1 or ER antibody 1:1000 and B-actin 1:5000 overnight at 4°C. For detection, the blot was incubated with LiCor IRDye secondary antibodies; 680LT goat anti-rabbit IgG and 800LT goat anti-mouse IgG both at 1:10,000 and visualized using an Odyssey scanner.

Quantitative real-time PCR analysis

Total RNA was extracted using the RNeasy mini kit (Qiagen) following the manufacturer's protocol. cDNA was prepared from 1μg RNA using Maxima reverse transcriptase (Fermentas) following the manufacturer's protocol. qPCR was performed in triplicate in 10 μl reactions with Power sybr mix (ABI) using Qiagen quantitect primers for *B2M*, *ACTB*, *CCNB1*, *CDC25B* and *FOXM1* and additional primers shown below. *ACTB* and *B2M* were used as housekeeping genes for normalization of the data. PCR conditions were; 95°C 10 min, 40 cycles of 95°C for 15 s and 60°C for 30 s followed by a dissociation curve (60-95°C). Relative expression levels were calculated using the delta delta C_T method.[1]

Microarray analysis

All data analyses were carried out using R with Bioconductor packages [2]. Raw intensity data from the array scanner were processed using the BASH [3] and HULK algorithms as implemented in the *beadarray* package [4].Briefly, Log2 transformation and quantile normalization of the data were performed across all samples. The LIMMA package was used to identify differentially expressed genes between the ligand treated and control samples. False discovery rate using the Benjamini and Hochberg's method was used to control for multiple testing [5] to identify statistically significant differentially expressed genes (FDR<0.01).

Chromatin immunoprecipitation

ChIP experiments carried as described previously [6] using primers detailed below.

ChIP-sequencing experiments

Single end 36-bp ChIP-seq data were generated by the Illumina analysis pipeline CASAVA 1.7 and OLB 1.9.4. Reads were aligned to the Human Reference Genome (assembly hg18, NCBI Build 36.6, March 2008) using bwa 0.6.1 [7] with default settings and reads that could not be confidently assigned to a unique genome position (i.e. with mapping quality mapq < 15) were removed (Table S4). In addition, reads overlapping regions known to accumulate unusually large number of reads in a nonspecific manner were excluded (excluded regions obtained from http://hgdownloadtest.cse.ucsc.edu/goldenPath/hg18/encodeDCC/wgEncodeMapability/wgEncodeDukeRegionsExcluded.bed). Read-enriched regions (*i.e.* binding sites) were identified with MACS 1.4.1 [8] using as control file a genomic input prepared from the same cell lines as the ChIP libraries. For the MCF7 cell line, a consensus set of binding sites was compiled by merging enriched regions detected in at least two libraries.

Co-immunoprecipitation

Experiments were performed using the Active Motif nuclear Co-immunoprecipitation kit following the manufacturer's protocol. In brief, MCF7 cells were harvested at 70% confluence in 15cm^2 dishes using PBS with phosphatase inhibitors at 4°C and spun at 1500rpm for 5 min at 4°C. Nuclear lysates were prepared by resuspending the cell pellets in 1X hypotonic buffer and incubating for 15 min on ice, following detergent addition the supernatant was centrifuged at 14,000xg for 30 s to pellet the nuclear fraction. The nuclear fraction was digested using complete digestion buffer with addition of the enzymatic shearing cocktail at 4°C for 2 h with end-to-end rotation. EDTA was added to give a final concentration of 10 mM and the cleared supernatant collected following centrifugation at 14,000xg for 10 min at 4°C. Protein concentration was measured by BCA (Pierce). Immunoprecipitation (IP) was performed using 400 μg protein per reaction with either 4 μg FOXM1 antibody (sc-502) or Rabbit IgG (Cell Signaling) using either the supplied low or high IP buffer supplemented with X1 protease inhibitor cocktail and in some samples 1mM DTT, in a final volume of 500 μl. Incubation was carried out O/N at 4°C with end-to-end rotation. Pull-down was performed by the addition of 50 μl pre-washed protein A magnetic beads (Invitrogen) for 1 h followed by X6 washes with IP buffer. After the final wash, beads were collected by centrifugation and resuspended in 15 μl of X1 Novex sample buffer (Invitrogen) and heated at 70°C for 10 min to release the bound proteins. Western blotting was performed as detailed above using antibodies for; FOXM1 (sc-502) 1:1000, ERα (Novacastra) 1:200 and CARM1 (sc-5421) 1:1000 with LiCor IRDye secondary antibodies; 680LT goat anti-rabbit IgG and 800LT goat anti-mouse IgG both at 1:10,000 and 680LT donkey anti-goat IgG at 1:15,000 and visualized using an Odyssey scanner

Survival analysis

The association between FOXM1 regulated genes and cancer relapse was conducted on the 209 ER positive patients from Wang *et al*. [9] (GEO accession GSE2034). This dataset contains gene expression (Affymetrix microarrays) and clinical data about tumor relapse and time to relapse.

For each probe, each patient was categorized as having 'high' expression, if the probe intensity was above the probe median intensity or 'low expression' otherwise. A Cox proportional hazards regression model was then fitted to the time to tumor relapse to test the significance of the expression category and to classify probes as leading to poor or good prognosis. Genes down-regulated by thiostrepton ($FDR < 0.01$) and having transcription start site no more than 50 kb from a FOXM1 binding site, were then tested for overrepresentation of genes significantly associated ($p < 0.01$) with poor prognosis when highly expressed (hypergeometric test). Genes down-regulated by thiostrepton and significantly associated to poor prognosis ($p < 0.01$) were used to cluster samples (hierarchical clustering) in the main groups to test for difference in time to relapse. The same gene set was used to cluster and test for difference in relapse the ER positive, untreated patients from Loi *et al*.[10] (GEO accession GSE2990). Survival analyses were performed with the R package survival.

Primer sequences:

qPCR primers

Primer Name	Forward	Reverse
AURKB	TACGGCCGACAGACGGCTCCA	AGCGGCTCATGAGGACAAGTGC
BIRC5	GCCAGATGACGACCCCATGCAA	TCGATGGCACGGCGCACTTT
CA12	CGTGGGCCTCAGTCTCCATC	GGCTGGCATTCTTGGCATCT
CCND1 nascent	TGCATCTACACCGACAACTCCA	CGGAGGAGCAGATATGTCAGAGG
CENPF	CGGCTGCGGGCAGTTTGAAT	AAATAAACTTGCTCTCGGGGACG
ESR1	CATGCCCTCTACACATTTTTCCC	TGATTGGTCTCGTCTGGGG
GREB1	CAAAGAATAACCTGTTGGCCCTGC	GACATGCCTGCGCTCTCATACTTA
GREB1 nascent	GTAGGAGTGTGCCCAGTGT	CCTGTTCAGCTGACCTCACA
MYC nascent	CAAAAATGAGGGGCTGTGTT	TTTGCCAAAAGTCCAAGAGG
NRIP	TCGCACTCACCACAGAAAAC	AGCCAAGCTCTTCTCCATGT
PLK1	TTCCCAAGCACATCAACCCCGT	AATGGTTGGGCGGGCAGTGG
pS2 nascent	CCTCACTGGACAGTTGCTGA	TGACACCAGGAAAACCACCAA
RARa	GGCTTCACCACCCCTCACCAT	AGCCCGTCCGAGAAGGTCAT
TFF1	GTGTCACGCCCTCCCAGT	GGACCCCACGAACGGTG
UBC nascent	TGAAGCTCCGGTTTTGAACT	CCAAAAACGGCCAGAATTTA
XBP1	GCGCCTCACGCACCTG	GCTGCTACTCTGTTTTTCAGTTTCC
XBP1 nascent	GCCCTGGTTGCTGAAGAGGA	CGCTGGGTCATGTCACTTGG

ChIP-qPCR

Primer Name	Forward	Reverse
AURKB promoter	GGGGTCCAAGGCACTGCTAC	GGGGCGGGAGATTTGAAAAG
BIRC5 promoter	CCATTAACCGCCAGATTTGA	TGTAGAGATGCGGTGGTCCT
CA12 Enhancer	GGAGGCGTAACCCCTGTGTG	ACGGCAAGGGACTTGCTGAC
CDC25B promoter	AAGAGCCCATCAGTTCCGCTTG	CCCATTTTACAGACCTGGACGC
CCNB1 promoter	CGCGATCGCCCTGGAAACGCA	CCCAGCAGAAACCAACAGCCGT
CENPF promoter	CACCTCCAGTAGAGGGGCTTG	TACCTCCACGCCTATTGGTC
ESR1	ATGGTCCGATTATGGGATGAT	CGGAAAAGGTCAAACTAACCTG

ESR1 enhancer	GAAACAGCCCCAAATCTCAA	TTGTAGCCAGCAAGCAAATG
FOXA1	CTGGGTTCACAGTGAGAATGAG	GGAGAAGATGGCAGTAACTTGG
FOXM1	CCGGAGCTTTCAGTTTGTTC	CGGAATGCCGAGACAAGG
GATA3	CCTCTGGGTGTTTTCAGACC	GGGTACGTGGGGTTTCTGTA
GREB1 enhancer	GAAGGGCAGAGCTGATAAC	GACCCAGTTGCCACACTTTT
MYB	AGCAATTTGGCACTTGGTCT	TAGCCTAGGGTGGCACAACT
MYC enhancer	GCTCTGGGCACACACATTGG	GGCTCACCCTTGCTGATGCT
NFkB1	GTTTGGAAGGGCAAAGGAAT	GTCACAGAGAGGTTTGGGAGTAA
NRIP enhancer	TGGCTCTCTCCTGCTGCTCA	CCAGACCCCTGTGTCCTTGC
NRIP1	AGGGTTTTCCGAGAAGTGCT	GTGACCGCAACCTGTTTCTT
PAPSS2	ATAGTTGGCGTGGAAGTTGG	CTGAGGCTTTAACGCAGGTC
PFKFB3 intron	GTGGCTTGGTCCTTGGTAGA	CTTGGCATTCACATCCATTG
PLK1 promoter	CCAGAGGGAGAAGATGTCCA	GTCGTTGTCCTCGAAAAAGC
PRMT8 intron	AAGGTCAGCTTTGCAAGGAA	TTTCTGAGTGTGCCAGCATC
RARa	CCCCACAGAGTTACTTGAGGTC	TAAAGCACTCCAAGGTAGGTG
RARa intron	GCTGGGTCCTCTGGCTGTTC	CCGGGATAAAGCCACTCCAA
RBL2	CCCCACAGAGTTAACTTGAGGTC	TAAAGCCACTCCAAGGTAGGTG
RDX	AAGAGTCAGGAAACCACAGGTC	GTGGAAGACCAACAGACTCACA
RERG	TTTCCAAACAGGTTTCCCTCTA	CTCTTACTGGAGGAAGGAACCA
TFF1 enhancer	AAACGCTGGCAACGACCTGT	ATGCTCTGCGCGGGCTAC
TOP2A	CGGAAAGCTTGGAAGAGATG	AGATTGGCAGTTCCTGGAGA
XBP1 enhancer	ATACTTGGCAGCCTGTGACC	GGTCCACAAAGCAGGAAAA
Actin control	AGCGCGGCTACAGCTTCA	CGTAGCACAGCTTCTCCTTAATGT
Cyclin D1 Control	TGCCACACACCAGTGACTTT	ACAGCCAGAAGCTCCAAAAA

Supplementary References

1. Livak KJ, Schmittgen TD: **Analysis of relative gene expression data using real-time quantitative PCR and the 2(-Delta Delta C(T)) Method.** *Methods* 2001, **25:**402-408.
2. Gentleman RC, Carey VJ, Bates DM, Bolstad B, Dettling M, Dudoit S, Ellis B, Gautier L, Ge Y, Gentry J, Hornik K, Hothorn T, Huber W, Iacus S, Irizarry R, Leisch F, Li C, Maechler M, Rossini AJ, Sawitzki G, Smith C, Smyth G, Tierney L, Yang JY, Zhang J: **Bioconductor: open software development for computational biology and bioinformatics.** *Genome biology* 2004, **5:R80.**
3. Cairns JM, Dunning MJ, Ritchie ME, Russell R, Lynch AG: **BASH: a tool for managing BeadArray spatial artefacts.** *Bioinformatics* 2008, **24:**2921-2922.
4. Dunning MJ, Smith ML, Ritchie ME, Tavare S: beadarray: **R classes and methods for Illumina bead-based data.** *Bioinformatics* 2007, **23:**2183-2184.
5. Klipper-Aurbach Y, Wasserman M, Braunspiegel-Weintrob N, Borstein D, Peleg S, Assa S, Karp M, Benjamini Y, Hochberg Y, Laron Z: **Mathematical formulae for the prediction of the residual beta cell function during the first two years of disease in children and adolescents with insulin-dependent diabetes mellitus.** *Medical hypotheses* 1995, **45:**486-490.
6. Schmidt D, Wilson MD, Spyrou C, Brown GD, Hadfield J, Odom DT: **ChIP-seq: using high-throughput sequencing to discover protein-DNA interactions.** *Methods* 2009, **48:**240-248.
7. Li H, Durbin R: **Fast and accurate short read alignment with Burrows-Wheeler transform.** *Bioinformatics* 2009, **25:**1754-1760.
8. Zhang Y, Liu T, Meyer CA, Eeckhoute J, Johnson DS, Bernstein BE, Nusbaum C, Myers RM, Brown M, Li W, Liu XS: **Model-based analysis of ChIP-Seq (MACS).** *Genome biology* 2008, **9:R137.**

9. Wang Y, Klijn JG, Zhang Y, Sieuwerts AM, Look MP, Yang F, Talantov D, Timmermans M, Meijer-van Gelder ME, Yu J, Jatkoe T, Berns EM, Atkins D, Foekens JA: **Gene-expression profiles to predict distant metastasis of lymph-node-negative primary breast cancer.** *Lancet* 2005, **365:**671-679.
10. Loi S, Haibe-Kains B, Desmedt C, Lallemand F, Tutt AM, Gillet C, Ellis P, Harris A, Bergh J, Foekens JA, Klijn JG, Larsimont D, Buyse M, Bontempi G, Delorenzi M, Piccart MJ, Sotiriou C: **Definition of clinically distinct molecular subtypes in estrogen receptor-positive breast carcinomas through genomic grade.** *Journal of clinical oncology : official journal of the American Society of Clinical Oncology* 2007, **25:**1239-1246.
11. Gyorffy B, Lanczky A, Eklund AC, Denkert C, Budczies J, Li Q, Szallasi Z: **An online survival analysis tool to rapidly assess the effect of 22,277 genes on breast cancer prognosis using microarray data of 1,809 patients.** *Breast cancer research and treatment* 2010**, 123:**725-731.

Table S1. Revigo analysis of FOXM1 binding sites

Revigo analysis of gene annotations of FOXM1 binding regions with FDR<0.01

Term_ID	Description	log10 p-value
GO:0008283	Cell proliferation	-3.7799
GO:0009725	Response to hormone stimulus	-3.1348
GO:0048545	Response to steroid hormone stimulus	-2.6753
GO:0016265	Death	-2.6068
GO:0051301	Cell division	-8.0482
GO:0060740	Prostate gland epithelium morphogenesis	-2.5781
GO:0060512	Prostate gland morphogenesis	-2.4472
GO:0071840	Cellular component organization or biogenesis	-2.5781
GO:0048285	Organelle fission	-4.5614
GO:0000279	M phase	-6.2514
GO:0000280	Nuclear division	-4.9042
GO:0007067	Mitosis	-4.9042
GO:0022403	Cell cycle phase	-5.0219
GO:0000087	M phase of mitotic cell cycle	-6.01
GO:0006915	Apoptotic process	-2.5781
GO:0012501	Programmed cell death	-2.6068
GO:0008219	Cell death	-2.6068
GO:0048523	Negative regulation of cellular process	-6.4818
GO:0045934	Negative regulation of nucleobase-containing compound metabolic process	-2.4424
GO:0045892	Negative regulation of transcription, DNA-dependent	-3.229
GO:0010629	Negative regulation of gene expression	-2.6068
GO:0010558	Negative regulation of macromolecule biosynthetic process	-2.5781
GO:0007049	Cell cycle	-7.3899
GO:0006357	Regulation of transcription from RNA polymerase II promoter	-2.8553
GO:0009719	Response to endogenous stimulus	-2.5424
GO:0048519	Negative regulation of biological process	-5.5884
GO:0050794	Regulation of cellular process	-3.7799
GO:0050789	Regulation of biological process	-2.6753
GO:0016043	Cellular component organization	-3.5373
GO:0006996	Organelle organization	-2.5781
GO:0022402	Cell cycle process	-5.6165
GO:0000278	Mitotic cell cycle	-4.5614
GO:0051726	Regulation of cell cycle	-2.9847
GO:0007010	Cytoskeleton organization	-2.5781

Table S2. Motif analysis of joint FOXM1/ER binding regions

Motif analysis using MEME with the JASPAR motif database on FOXM1 binding regions in MCF7 showing the top 50 motifs identified in regions of joint binding with ERα and comparing the p-value to that in regions of FOXM1 only binding. (NA indicates motif not found)

Term_ID	Factor	Family	p-value	
			Joint FOXM1 /ER regions	FOXM1 regions
MA0148.1	FOXA1	Forkhead domain family	4.03E-303	7.62E-38
MA0047.2	Foxa2	Forkhead domain family	3.34E-272	8.09E-25
MA0446.1	fkh	Forkhead domain family	4.27E-216	5.14E-24
MA0047.1	Foxa2	Forkhead domain family	9.84E-150	1.35E-09
MA0099.1	Fos	Leucine zipper family	2.27E-131	8.45E-32
MA0297.1	FKH2	Forkhead domain family	4.59E-115	6.44E-13
MA0303.1	GCN4	Leucine zipper family	4.24E-107	8.34E-28
MA0030.1	FOXF2	Forkhead domain family	6.48E-106	7.47E-12
MA0099.2	AP1	Leucine zipper family	1.71E-101	8.45E-26
MA0296.1	FKH1	Forkhead domain family	9.72E-97	8.30E-09
MA0042.1	FOXI1	Forkhead domain family	2.03E-92	2.02E-08
MA0041.1	Foxd3	Forkhead domain family	3.48E-79	1.53E-07
MA0458.1	slp1	Forkhead domain family	3.04E-75	4.51E-09
MA0272.1	ARG81	Fungal Zn cluster	1.08E-70	2.63E-17
MA0031.1	FOXD1	Forkhead domain family	4.53E-61	1.13E-06
MA0035.2	Gata1	GATA domain family	2.12E-60	NA
MA0112.2	ESR1	Hormone-Nuclear receptor family	4.97E-53	NA
MA0003.1	TFAP2A	Helix-Loop-Helix family	5.88E-53	NA
MA0258.1	ESR2	Hormone-Nuclear receptor family	2.03E-50	NA
MA0157.1	FOXO3	Hormone-Nuclear receptor family	5.79E-46	NA
MA0040.1	Foxq1	Forkhead domain family	6.52E-34	NA
MA0317.1	HCM1	Forkhead domain family	1.36E-33	NA
MA0160.1	NR4A2	Hormone-Nuclear receptor family	2.61E-31	NA
MA0140.1	Tal1::Gata1	Helix-Loop-Helix family	4.64E-27	NA
MA0112.1	ESR1	Hormone-Nuclear receptor family	7.02E-27	NA
MA0071.1	RORA_1	Hormone-Nuclear receptor family	1.10E-25	NA
MA0419.1	YAP7	Leucine zipper family	2.82E-24	7.02E-06
MA0149.1	EWSR1-FLI1	Ets	8.15E-23	NA
MA0150.1	NFE2L2	Leucine zipper family	2.19E-22	NA
MA0039.2	Klf4	BetaBetaAlpha-zinc finger	3.03E-21	1.04E-12
MA0406.1	TEC1	Homeo	3.83E-19	NA
MA0090.1	TEAD1	Homeo	6.97E-16	NA
MA0070.1	PBX1	Homeo	1.39E-15	NA
MA0013.1	br_Z4	BetaBetaAlpha-zinc finger	2.49E-15	NA
MA0002.2	RUNX1	Runt	3.86E-15	NA
MA0118.1	Macho-1	BetaBetaAlpha-zinc finger	7.77E-15	1.70E-10
MA0119.1	TLX1::NFIC	Homeo::Nuclear Factor I-CCAAT-binding	7.63E-14	NA
MA0141.1	Esrrb	Hormone-Nuclear receptor family	1.99E-13	NA
MA0045.1	HMG-I/Y	High Mobility group	4.69E-13	0.0004732

MA0443.1	btd	BetaBetaAlpha-zinc finger	2.39E-12	3.71E-13
MA0079.2	SP1	BetaBetaAlpha-zinc finger	8.86E-12	4.91E-08
MA0152.1	NFATC2	Rel	1.85E-11	4.48E-07
MA0164.1	Nr2e3	Hormone-Nuclear receptor family	1.02E-09	NA
MA0026.1	Eip74EF	Ets	1.88E-09	1.05E-11
MA0300.1	GAT1	GATA domain family	9.03E-09	NA
MA0423.1	YER130C	Leucine zipper family	1.37E-08	NA
MA0084.1	SRY	High Mobility group	1.75E-08	NA
MA0372.1	RPH1	BetaBetaAlpha-zinc finger	3.11E-08	0.0002311
MA0144.1	Stat3	Stat	6.66E-08	NA
MA0306.1	GIS1	BetaBetaAlpha-zinc finger	1.08E-07	NA

Table S3. Motif analysis of FOXM1 only binding regions

Motifs identified using MEME with the JASPAR motif database from regions where FOXM1 is bound but not ERα in MCF7 cells, only the unique motifs that are not present in the joint FOXM1/ER binding regions are shown with the corresponding p-value.

**Term_ID**	**Factor**	**DNA binding domain**	**p-value**
			**FOXM1 only regions**
MA0060.1	NFYA	NFY CCAAT-binding	1.43E-18
MA0316.1	HAP5	NFY CCAAT-binding	7.67E-13
MA0139.1	CTCF	BetaBetaAlpha-zinc finger	3.60E-10
MA0076.1	ELK4	Ets	1.66E-09
MA0062.1	GABPA	Ets	1.85E-09
MA0028.1	ELK1	Ets	2.95E-07
MA0450.1	hkb	BetaBetaAlpha-zinc finger	4.09E-06
MA0275.1	ASG1	Fungal Zn cluster	3.11E-05
MA0080.2	SPI1	Ets	7.81E-05
MA0096.1	bZIP910	Leucine zipper family	0.000101
MA0162.1	Egr1	BetaBetaAlpha-zinc finger	0.0001551

Table S4. Motif analysis of FOXM1 peaks in MDA-MB-231 vs MCF7 cells

Motif analysis performed using MEME with the JASPAR motif database on the FOXM1 binding regions in MDA-MB-231 cells showing the top 50 motifs identified comparing the p-value to regions of FOXM1 only binding in MCF7 cells. (NA indicates motif not found).

Term_ID	Factor	Family	p-value	
			MDA-MB-231	MCF7 (FOXM1 only regions)
MA0099.1	Fos	Leucine zipper family	8.56E-108	8.45E-32
MA0062.2	GABPA	Ets	7.03E-106	2.90E-12
MA0099.2	AP1	Leucine zipper family	1.23E-89	8.45E-26
MA0026.1	Eip74EF	Leucine zipper family	2.14E-88	1.05E-11
MA0303.1	GCN4	Ets	4.69E-83	8.34E-28
MA0156.1	FEV	Ets	1.10E-75	2.53E-07
MA0062.1	GABPA	Ets	2.28E-71	1.85E-09
MA0039.2	Klf4	BetaBetaAlpha-zinc finger	2.15E-68	1.04E-12
MA0272.1	ARG81	Fungal Zn cluster	2.27E-66	2.63E-17
MA0118.1	Macho-1	BetaBetaAlpha-zinc finger	6.46E-61	1.70E-10
MA0443.1	btd	BetaBetaAlpha-zinc finger	8.99E-60	3.71E-13
MA0076.1	ELK4	Ets	1.39E-59	1.66E-09
MA0136.1	ELF5	Ets	1.17E-58	1.46E-05
MA0060.1	NFYA	NFY CCAAT-binding	1.59E-47	1.43E-18
MA0283.1	CHA4	Fungal Zn cluster	2.21E-45	9.57E-06
MA0079.2	SP1	BetaBetaAlpha-zinc finger	2.48E-42	4.91E-08
MA0450.1	hkb	BetaBetaAlpha-zinc finger	1.37E-39	4.91E-08
MA0144.1	Stat3	Stat	5.39E-37	NA
MA0028.1	ELK1	Ets	1.86E-35	2.95E-07
MA0149.1	EWSR1-FLI1	Ets	4.38E-32	NA
MA0002.2	RUNX1	Runt	6.98E-32	NA
MA0080.2	SPI1	Ets	1.75E-31	7.81E-05
MA0419.1	YAP7	Leucine zipper family	3.53E-31	7.02E-06
MA0314.1	HAP3	NFY CCAAT-binding	5.85E-31	1.24E-14
MA0285.1	CRZ1	BetaBetaAlpha-zinc finger	7.54E-29	NA
MA0004.1	Arnt	Helix-Loop-Helix family	1.64E-28	NA
MA0093.1	USF1	Helix-Loop-Helix family	1.64E-28	NA
MA0104.1	Mycn	Helix-Loop-Helix family	1.64E-28	NA
MA0275.1	ASG1	Fungal Zn cluster	1.74E-27	3.11E-05
MA0080.1	SPI1	Ets	6.21E-27	NA
MA0098.1	ETS1	Ets	6.21E-27	NA
MA0096.1	bZIP910	Leucine zipper family	3.70E-25	0.000101
MA0137.2	STAT1	Stat	3.62E-24	NA
MA0281.1	CBF1	Helix-Loop-Helix family	8.29E-24	NA
MA0410.1	UGA3	Fungal Zn cluster	6.34E-22	NA
MA0316.1	HAP5	NFY CCAAT-binding	1.60E-21	7.67E-13
MA0286.1	CST6	Leucine zipper family	2.19E-21	NA
MA0242.1	run::Bgb	Runt	2.68E-21	NA
MA0105.1	NFKB1	Rel	4.00E-20	NA
MA0058.1	MAX	Helix-Loop-Helix family	1.81E-19	NA
MA0332.1	MET28	Leucine zipper family	3.08E-19	NA
MA0315.1	HAP4	NFY CCAAT-binding	5.55E-18	9.56E-09

MA0399.1	SUT1	Fungal Zn cluster	6.86E-18	NA
MA0002.1	RUNX1	Runt	2.40E-17	NA
MA0310.1	HAC1	Leucine zipper family	2.74E-17	NA
MA0162.1	Egr1	BetaBetaAlpha-zinc finger	6.49E-17	NA
MA0067.1	Pax2	Homeo	7.31E-16	NA
MA0414.1	XBP1	Rel	8.19E-16	NA
MA0128.1	EmBP-1	Leucine zipper family	9.48E-16	NA
MA0409.1	TYE7	Helix-Loop-Helix family	1.72E-15	NA

Table S5. ChIP-Seq libraries used for mapping FOXM1 binding sites.

Library ID	Cell line	ChIP	Treatment	Replicate	N. reads	N. Aligned	MAPQ > 15
001	MCF-7	foxm1	DMSO	A	31367142	29131863	24368185
002	MCF-7	foxm1	Thiostrepton	A	38487095	37575186	31447915
003	MCF-7	foxm1	DMSO	B	28936359	28280835	22635935
004	MCF-7	foxm1	Thiostrepton	B	33893474	33276954	26664385
011	MCF-7	foxm1	DMSO	C	15626190	14819797	12382305
012	MCF-7	foxm1	Thiostrepton	C	13930259	13242204	11091548
017	MCF-7	foxm1	DMSO	D	33000000	32037483	26907404
018	MCF-7	foxm1	Thiostrepton	D	30955447	30396177	25480949
379	MCF-7	input			28226691	27799300	22254141
005	MDA-MB-231	foxm1	DMSO	a	28215261	27397904	21470698
006	MDA-MB-231	foxm1	Thiostrepton	a	33608493	32764794	26622102
007	MDA-MB-231	foxm1	DMSO	b	32492981	31479855	25368094
008	MDA-MB-231	foxm1	Thiostrepton	b	31168350	30162982	24443020
168	MDA-MB-231	input			15867588	15390320	12318689

Table S6. Motif analysis of thiostrepton regulated peaks

Motifs identified using SeqPos motif tool with Cistrome motif database (p-value<0.001) in peaks identified by DBA as differentially bound by thiostrepton treatment

THIOSTREPTON INCREASED PEAKS		THIOSTREPTON DECREASED PEAKS	
**Term_ID**	**Factor**	**Term_ID**	**Factor**
M00490	BACH2	MA0148	FOXA1
MC00019	FOS	MA0031	FOXD1
MA00028	JUN	MC00023	FOXA2
MC00044	SMARCC1		

Table S7. Differentially expressed genes from microarray

Differentially expressed gene lists from microarray analysis for DMSO versus thiostrepton treated MCF7 cells.

Upregulated genes

Gene	Ref Seq	Description	LogFC	*P*-Value
HSPA6	NM_002155.3	heat shock 70kDa protein 6 (HSP70B')	7.34	9.91E-32
HSPA6	NM_002155.3	heat shock 70kDa protein 6 (HSP70B')	5.67	3.72E-27
LOC652750	XR_038244.1		5.35	3.98E-29
HSPA7	NR_024151.1	heat shock 70kDa protein 7 (HSP70B)	5.02	7.97E-28
HMOX1	NM_002133.1	heme oxygenase (decycling) 1	4.73	1.88E-26
ZFAND2A	NM_182491.1	zinc finger, AN1-type domain 2A	3.62	2.37E-26
ATF	NM_001040619.1	activating transcription factor 3	3.56	4.24E-23
UBC	NM_021009.3	ubiquitin C	3.56	3.61E-25
PPP1R15A	NM_014330.2	protein phosphatase 1, regulatory subunit 15A	3.55	4.98E-25
IER5	NM_016545.4	immediate early response 5	3.43	1.17E-24

Downregulated genes

Gene	Ref Seq	Description	LogFC	*P*-Value
DKK1	NM_012242.2	dickkopf 1 homolog (Xenopus laevis)	-3.08	5.27E-23
IRS1	NM_005544.1	insulin receptor substrate 1	-2.15	3.43E-18
CXCR7	NM_001047841.1	chemokine (C-X-C motif) receptor 7	-1.82	1.13E-16
CSTF3	NM_001326.2	cleavage stimulation factor, 3' pre-RNA, subunit 3, 77kDa	-1.81	4.92E-18
ARID5B	NM_032199.1	AT rich interactive domain 5B (MRF1-like)	-1.79	2.49E-19
NRP1	NM_003873.4	neuropilin 1	-1.67	1.18E-16
PUS7	NM_019042.3	pseudouridylate synthase 7 homolog (S. cerevisiae)	-1.52	3.14E-16
FAM120B	NM_032448.1	family with sequence similarity 120B	-1.46	1.86E-16
C16ORF53	NM_024516.2	chromosome 16 open reading frame 53	-1.276	2.35E-16
TCFL5	NM_006602.2	transcription factor-like 5 (basic helix-loop-helix)	-1.24	3.18E-16

Table S8. DBA regions for validation

Binding regions selected for ChIP-PCR validation from DBA analysis with FDR<0.05 correlated with differential gene expression from microarray analysis (FDR<0.01)

GENE	Peak Log FC	Peak FDR	Array Log FC	Array p-value
CA12	-0.66	2.52E-02	-1.18	3.55E-12
ESR1	-1.14	1.27E-05	-0.85	2.92E-09
FOXA1	-0.94	7.31E-03	-1.02	1.43E-12
GATA3	-1.66	1.19E-11	-1.47	5.54E-15
GREB1	-0.88	1.74E-04	-0.98	3.16E-10
MIPOL1	-1.11	1.62E-04	-0.30	8.70E-04
MYC	-0.88	4.91E-04	-0.33	2.04E-03
MYB	-0.72	1.26E-02	-1.06	3.21E-11
NFKB1	-0.94	1.70E-02	-0.53	1.05E-04
NRIP1	-1.09	1.22E-04	-0.57	1.21E-06
PAPSS2	-0.94	7.47 E-03	-0.50	4.47E-06
RARa	-0.59	4.79E-02	-0.58	1.22E-07
RBL2	-0.76	2.59E-02	-0.58	2.11E-07
RDX	-1.43	2.56E-07	-0.99	5.66E-11
RERG	-0.97	1.51E-04	-0.84	8.51E-09
TOP2A	-0.60	3.08E-02	-0.83	1.03E-08
XBP-1	-0.91	3.24E-04	-0.64	5.66E-09

Table S9. GSEA analysis of Thiostrepton downregulated genes

Gene set enrichment Analysis (GSEA) using genes downregulated by thiostrepton treatment with a FOXM1 binding peak within 50kb of TSS (198 unique genes). The top ten significant overlapping sets in the Molecular Signature database are shown with the corresponding p values calculated by hypergeometric distribution.

Gene set list	Description	Genes in set	Genes in overlap	p value
CREIGHTON_ENDOCRINE_ THERAPY_ RESISTANCE_1	The 'group 1 set' of genes associated with acquired endocrine therapy resistance in breast tumors expressing ESR1 and ERBB2 [Gene ID=2099, 2064].	526	29	0.00E+00
GOZGIT_ESR1_ TARGETS_DN	Genes down-regulated in TMX2-28 cells (breast cancer) which do not express ESR1 [Gene ID=2099]) compared to the parental MCF7 cells which do.	776	44	0.00E+00
MASSARWEH_TAMOXIFEN _RESISTANCE_DN	Genes down-regulated in breast cancer tumours (formed by MCF-7 xenografts) resistant to tamoxifen [PubChem=5376].	253	20	3.33E-16
SMID_BREAST_CANCER_ BASAL_DN	Genes down-regulated in basal subtype of breast cancer samples.	713	28	2.05E-14
FRASOR_RESPONSE_TO_ ESTRADIOL_UP	Genes up-regulated in MCF-7 cells (breast cancer) by estradiol (E2) [PubChem=5757].	32	8	2.31E-11
CREIGHTON_ENDOCRINE_ THERAPY_RESISTANCE_4	The 'group 4 set' of genes associated with acquired endocrine therapy resistance in breast tumours expressing ESR1 but not ERBB2 [Gene ID=2099, 2064].	308	16	1.86E-10
RIGGINS_TAMOXIFEN_ RESISTANCE_DN	Genes down-regulated SUM44/LCCTam cells (breast cancer) resistant to 4-hydroxytamoxifen [PubChem=63062] relative to the parental SUM44 cells sensitive to the drug.	221	14	1.99E-10
DOANE_BREAST_CANCER_ ESR1_UP	Genes changed in breast cancer samples according to the ESR1 [Gene ID=2099] status: ER positive vs ER negative tumours.	114	11	2.09E-10
STEIN_ESR1_TARGETS	Genes regulated by ESR1 [Gene ID=2099] in MCF-7 cells (breast cancer).	85	9	4.18E-09
FARMER_BREAST_ CANCER_ BASAL_VS_LULMINAL	Genes which best discriminated between two groups of breast cancer according to the status of ESR1 and AR [Gene ID=2099, 367]: basal (ESR1- AR-) and luminal (ESR1+ AR+).	335	15	5.29E-09
XU_GH1_AUTOCRINE_ TARGETS_DN	Genes down-regulated in MFCF-7 cells (breast cancer) upon stable autocrine expression of HG1 [Gene ID=2688].	10	124	8.20E-09

Table S10. GSEA analysis of Thiostrepton upregulated genes

Gene set enrichment Analysis (GSEA) using genes upregulated by thiostrepton treatment with a FOXM1 binding peak within 50kb of TSS (111 unique genes). The top ten significant overlapping sets in the Molecular Signature database are shown with the corresponding p values calculated by hypergeometric distribution.

Gene set list	Description	Genes in set	Genes in overlap	p value
BUYTAERT_PHOTODYNAMIC_THERAPY_STRESS_UP	Genes up-regulated in T24 (bladder cancer) cells in response to the photodynamic therapy (PDT) stress.	824	34	0.00E+00
GARGALOVIC_RESPONSE_TO_OXIDIZED_PHOSPHOLIPIDS_BLUE_UP	Genes from the blue module which are up-regulated in HAEC cells (primary aortic endothelium) after exposure to the oxidized 1-palmitoyl-2-arachidonyl-sn-3-glycerophosphorylcholine (oxPAPC).	133	13	2.11E-15
TIEN_INTESTINE_PROBIOTICS_24HR_DN	Genes down-regulated in Caco-2 cells (intestinal epithelium) after coculture with the probiotic bacteria L. casei for 24h.	221	12	2.88E-11
HELLER_HDAC_TARGETS_SILENCED_BY_METHYLATION_DN	Genes down-regulated in multiple myeloma (MM) cell lines treated with both decitabine [PubChem=451668] TSA [PubChem=5562].	244	12	9.07E-11
CYTOPLASM	Genes annotated by the GO term GO:0005737. All of the contents of a cell excluding the plasma membrane and nucleus, but including other subcellular structures.	2066	29	1.42E-10
PODAR_RESPONSE_TO_ADAPHOSTIN_UP	Up-regulated genes in MM1.S cells (multiple myeloma) treated with adaphostin [PubChem=387042], a tyrosine kinase inhibitor with anticancer properties.	151	10	1.98E-10
NUYTTEN_EZH2_TARGETS_UP	Genes up-regulated in PC3 cells (prostate cancer) after knockdown of EZH2 [Gene ID=2146] by RNAi.	974	20	2.86E-10
MACROMOLECULE_CATABOLIC_PROCESS	Genes annotated by the GO term GO:0009057. The chemical reactions and pathways resulting in the breakdown of a macromolecule, any large molecule including proteins, nucleic acids and carbohydrates.	135	9	1.56E-09
DIAZ_CHRONIC_MEYLOGENOUS_LEUKEMIA_UP	Genes up-regulated in CD34+ [Gene ID=947] cells isolated from bone marrow of CML (chronic myelogenous leukemia) patients, compared to those from normal donors.	1398	22	4.65E-09

Table S11. Thiostrepton regulated genes associated with prognosis

Gene list of thiostrepton down-regulated genes containing FOXM1 binding site (±50kb TSS) significantly correlated with prognosis in breast cancer patient datasets.

Gene ID	Entrez Gene Name	Location	Type
ABCC5	ATP-binding cassette, sub-family C (CFTR/MRP), member 5	Plasma Membrane	transporter
ASPM	asp (abnormal spindle) homolog, microcephaly associated (Drosophila)	Nucleus	other
AURKA	aurora kinase A	Nucleus	kinase
BUB1B	budding uninhibited by benzimidazoles 1 homolog beta (yeast)	Nucleus	kinase
CCDC99	coiled-coil domain containing 99	Nucleus	other
CCNB1	cyclin B1	Cytoplasm	other
CDC20	cell division cycle 20 homolog (S. cerevisiae)	Nucleus	other
CENPE	centromere protein E, 312kDa	Nucleus	other
CEP55	centrosomal protein 55kDa	Cytoplasm	other
CKAP2	cytoskeleton associated protein 2	Cytoplasm	other
DEPDC1	DEP domain containing 1	Nucleus	transcription regulator
DLGAP5	discs, large (Drosophila) homolog-associated protein 5	Nucleus	phosphatase
DPY19L4	dpy-19-like 4 (C. elegans)	unknown	other
E2F8	E2F transcription factor 8	Nucleus	other
FANCI	Fanconi anemia, complementation group I	Nucleus	other
FBXO5	F-box protein 5	Nucleus	enzyme
GEMIN6	gem (nuclear organelle) associated protein 6	Nucleus	other
KIAA0391	KIAA0391	unknown	other
KIAA0406	TELO2 interacting protein 1	unknown	other
KIF11	kinesin family member 11	Nucleus	other
KIF14	kinesin family member 14	Cytoplasm	other
LEPREL4	leprecan-like 4	Nucleus	other
LMNB1	lamin B1	Nucleus	other
MAD2L1	MAD2 mitotic arrest deficient-like 1 (yeast)	Nucleus	other
METTL2A	methyltransferase like 2A	unknown	other
NEIL3	nei endonuclease VIII-like 3 (E. coli)	Nucleus	enzyme
NMU	neuromedin U	Extracellular Space	other
OIP5	Opa interacting protein 5	Nucleus	other
PIAS3	protein inhibitor of activated STAT, 3	Nucleus	transcription regulator
RBL1	retinoblastoma-like 1 (p107)	Nucleus	other
RFC4	replication factor C (activator 1) 4, 37kDa	Nucleus	other
RPA3	replication protein A3, 14kDa	Nucleus	other
RPRD1A	regulation of nuclear pre-mRNA domain containing 1A	unknown	other
RRM2	ribonucleotide reductase M2	Nucleus	enzyme
SURF2	surfeit 2	unknown	other
TMEM134	transmembrane protein 134	unknown	kinase
TRIP13	thyroid hormone receptor interactor 13	Cytoplasm	transcription regulator
TTF2	transcription termination factor, RNA polymerase II	Nucleus	transcription regulator

Figure S1. Motif and CEAS analysis of FOXM1 binding sites in MCF7 cells
(A) Motif analysis of FOXM1 binding regions identified a set of motifs only in the regions of FOXM1 binding only and not in the regions bound by both FOXM1 and ER in MCF7 cells. CEAS analysis (B) showing genomic FOXM1 binding in regions joint binding with ER compared to FOXM1 only regions.

A

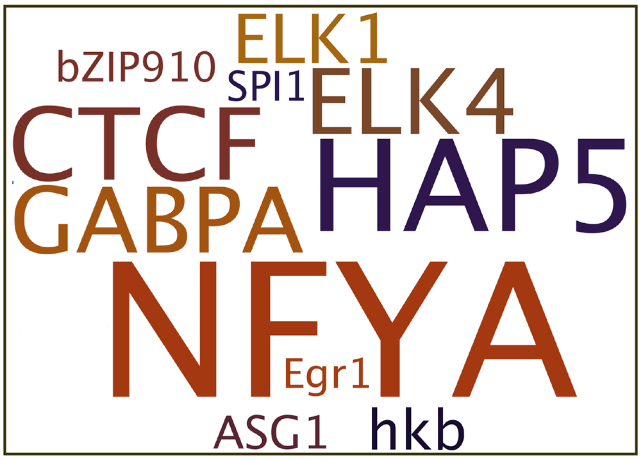

B

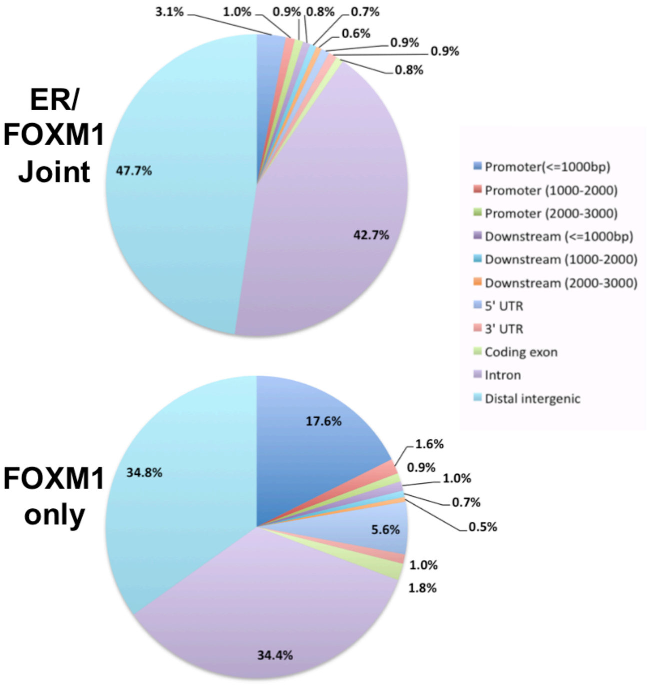

Figure S2. qPCR following fulvestrant treatment of MCF7 cells
qPCR was used to measure expression levels of genes downstream of regions with FOXM1 only binding or co-binding of ERα and FOXM1 following treatment of MCF7 with fulvestrant for 3 h. Data shows mean of triplicate experiments ±SD. (*) *P*<0.05, (**) *P*<0.01, (***) *P*<0.001.

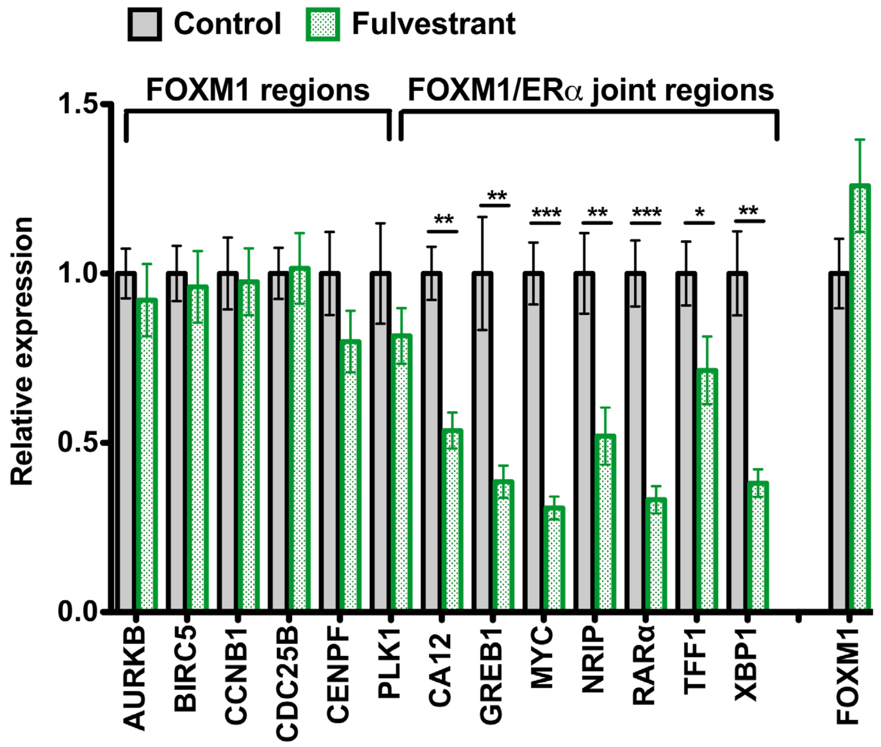

Figure S3. FOXM1 ChIP-qPCR of MCF7 cells treated with Fulvestrant/MG132
(A) Western blot of ER and FOXM1 levels in MCF7 cells treated with fulvestrant (10nM) or MG132 (3μM) for 3 h. (**B)** ChIP-PCR results for MCF7 treated with fulvestrant or MG132 for 3h using primers for regions of joint binding of FOXM1/ER or FOXM1 only. Data representative of triplicate experiments ±SD. (*) $P<0.05$, (**) $P<0.01$, (***) $P<0.001$.

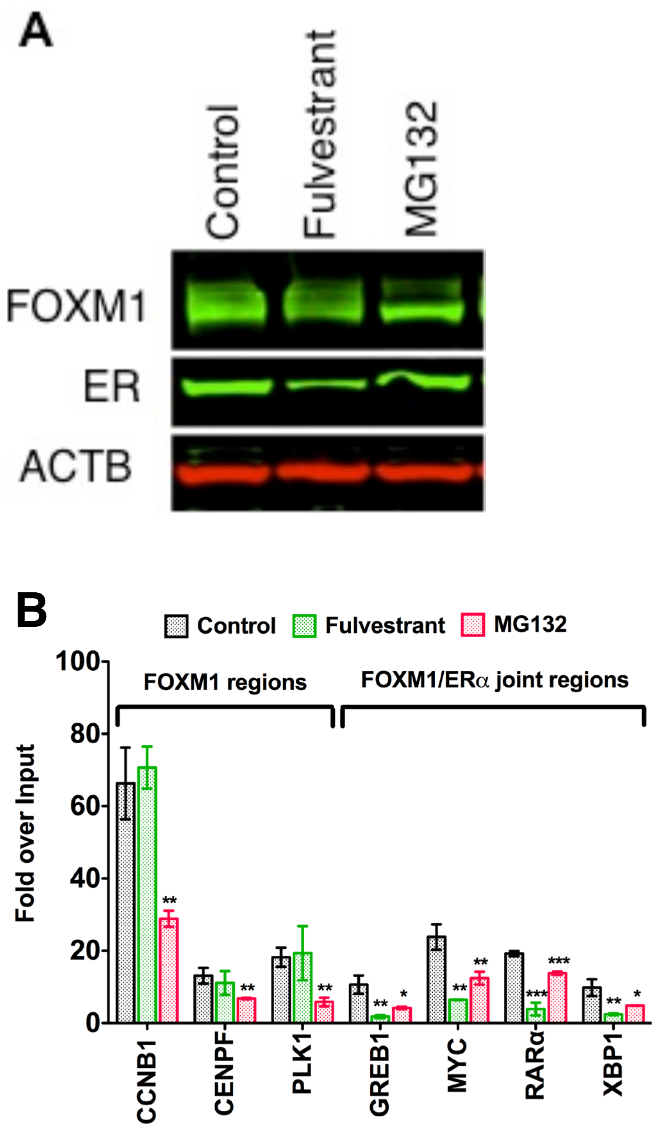

Figure S4. CEAS analysis of FOXM1 binding in MCF7 vs MDA-MB-231

CEAS analysis showing genomic FOXM1 binding in regions of overlap in MDA-MB-231 and MCF7 cells.

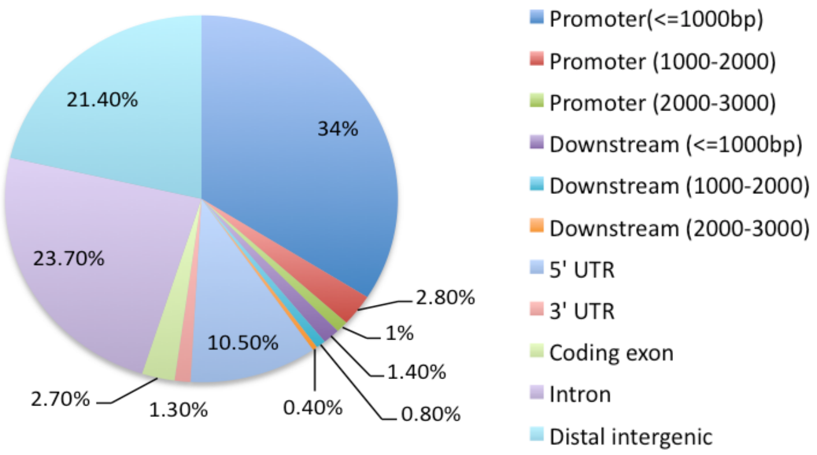

Figure S5. FOXM1 ChIP-qPCR

ChIP-qPCR of FOXM1 binding using two additional FOXM1 antibodies (GTX102170 and GTX100276) showing similar enrichment profile to sc-502 at ER co-bound sites.

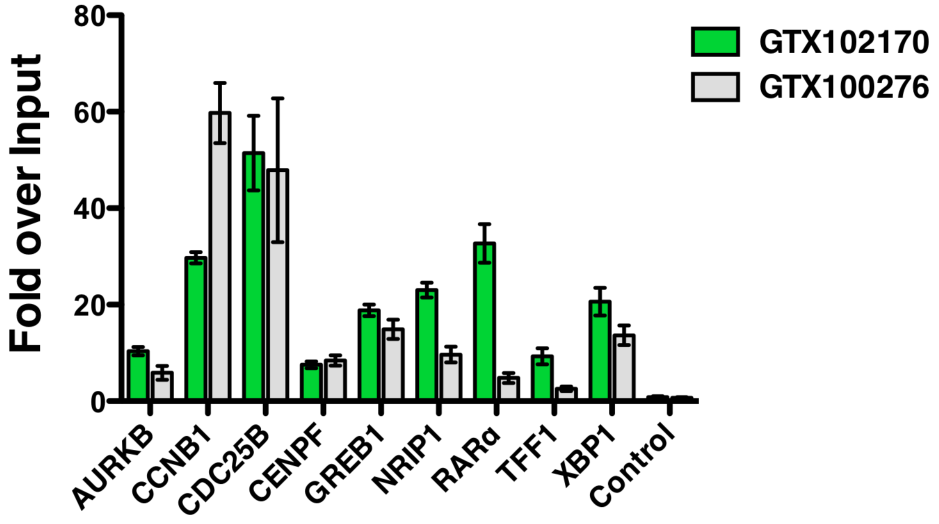

Figure S6. CEAS analysis of FOXM1 DBA regions

CEAS analysis of FOXM1 binding in peaks identified from DBA as differentially bound after treatment of MCF7 cells with thiostrepton compared to the DMSO control. This compares the peak distribution in the increased/decreased binding sites compared to the whole genome.

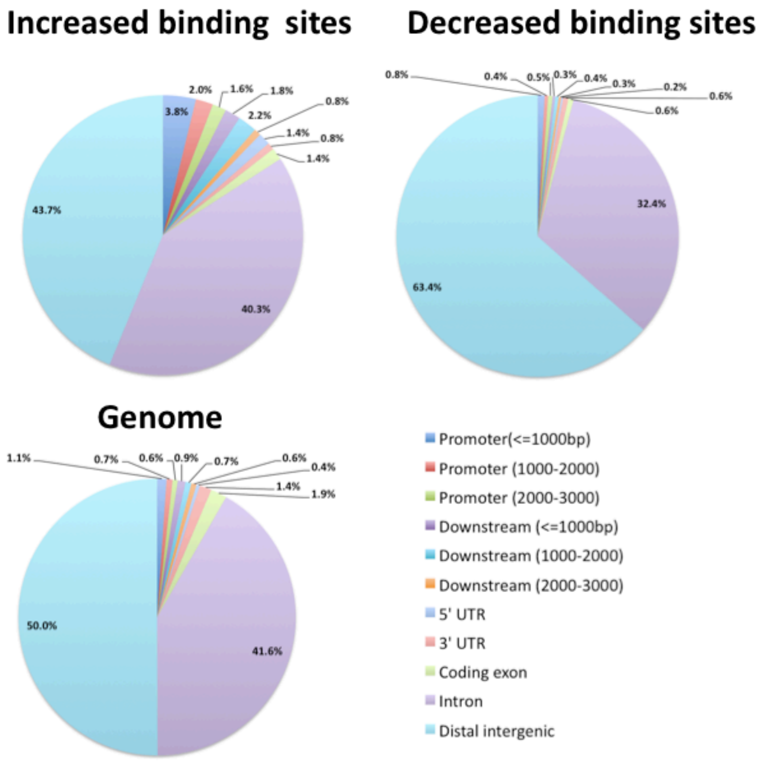

Figure S7. GSEA analysis of Thiostrepton downregulated genes

GSEA results comparing genes with FOXM1 binding peaks within 50 kb of TSS downregulated by thiostrepton treatment (198 unique genes) with gene signature sets present in the MSigDB. Genes from the top 10 most significantly overlapped datasets are shown with the shaded blocks indicating presence in dataset. Only genes overlapping with one or more datasets shown (76 genes)

gene_symbol	CREIGHTON_ENDOCRINE_THERAPY_RESISTANCE_1	CREIGHTON_ENDOCRINE_THERAPY_RESISTANCE_4	DOANE_BREAST_CANCER_ESR1_UP	FARMER_BREAST_CANCER_BASAL_VS_LULMINAL	FRASOR_RESPONSE_TO_ESTRADIOL_UP	GOZGIT_ESR1_TARGETS_DN	MASSARWEH_TAMOXIFEN_RESISTANCE_DN	RIGGINS_TAMOXIFEN_RESISTANCE_DN	SMID_BREAST_CANCER_BASAL_DN	STEIN_ESR1_TARGETS	description
ADCY1	■					■			■		adenylate cyclase 1 (brain)
APBB2	■			■		■	■	■	■		amyloid beta (A4) precursor protein-binding, family B, member 2 (Fe65-like)
ATIC	■										5-aminoimidazole-4-carboxamide ribonucleotide formyltransferase/IMP cyclohydrolase
C1QTNF6	■										C1q and tumor necrosis factor related protein 6
C6ORF211	■		■	■		■	■		■		chromosome 6 open reading frame 211
CALCR	■	■			■	■				■	calcitonin receptor
CAP2	■					■				■	CAP, adenylate cyclase-associated protein, 2 (yeast)
CCDC83	■					■					coiled-coil domain containing 83
CDC25B	■										cell division cycle 25B
COL4A5	■	■				■	■		■		collagen, type IV, alpha 5 (Alport syndrome)
EGR3	■	■			■	■	■		■		early growth response 3
ESR1	■		■	■		■	■	■	■		estrogen receptor 1
FLJ45983	■					■					-
GATA3	■		■	■		■			■		GATA binding protein 3
GREB1	■	■	■	■	■	■	■	■	■	■	-
H2AFJ	■										H2A histone family, member J
HEY2	■										hairy/enhancer-of-split related with YRPW motif 2
LYPD6	■										LY6/PLAUR domain containing 6
MYC	■	■		■							v-myc myelocytomatosis viral oncogene homolog (avian)
NRCAM	■					■	■	■			neuronal cell adhesion molecule
PKIB	■	■				■	■				protein kinase (cAMP-dependent, catalytic) inhibitor beta
PRSS23	■	■			■	■	■		■	■	protease, serine, 23
RAB31	■	■			■		■	■		■	RAB31, member RAS oncogene family
RERG	■	■				■	■				RAS-like, estrogen-regulated, growth inhibitor
RHOBTB3	■	■				■	■				Rho-related BTB domain containing 3
SYT1	■		■			■	■	■	■		synaptotagmin I
SYTL2	■					■			■		synaptotagmin-like 2
TMEM164	■	■					■				transmembrane protein 164
UBE2T	■										ubiquitin-conjugating enzyme E2T (putative)
ADCY9		■			■	■	■		■	■	adenylate cyclase 9
CBX2		■					■				chromobox homolog 2 (Pc class homolog, Drosophila)
FGD3		■									FYVE, RhoGEF and PH domain containing 3
MYB		■	■	■			■		■		v-myb myeloblastosis viral oncogene homolog (avian)
RFX5		■									regulatory factor X, 5 (influences HLA class II expression)
BMPR1B			■			■			■		bone morphogenetic protein receptor, type IB
FOXA1			■	■		■			■		forkhead box A1
SEMA3C			■	■		■		■	■		sema domain, immunoglobulin domain (Ig), short basic domain, secreted, (semaphorin) 3C
SLC44A4			■	■					■		solute carrier family 44, member 4
XBP1			■	■		■			■		X-box binding protein 1
GPD1L				■					■		glycerol-3-phosphate dehydrogenase 1-like
NT5C2				■							5'-nucleotidase, cytosolic II
RARA				■		■			■		retinoic acid receptor, alpha
STEAP3				■				■			STEAP family member 3
RASGRP1					■	■					RAS guanyl releasing protein 1 (calcium and DAG-regulated)
TOP2A					■		■				topoisomerase (DNA) II alpha 170kDa
ANKRD50						■					ankyrin repeat domain 50
ARID5B						■		■			AT rich interactive domain 5B (MRF1-like)
ASB9						■					ankyrin repeat and SOCS box-containing 9
C10ORF81						■					chromosome 10 open reading frame 81
C3ORF57						■					chromosome 3 open reading frame 57
C9ORF150						■					chromosome 9 open reading frame 150
CMBL						■					carboxymethylenebutenolidase homolog (Pseudomonas)
GULP1						■		■			GULP, engulfment adaptor PTB domain containing 1
NEBL						■		■	■		nebulette
NR5A2						■					nuclear receptor subfamily 5, group A, member 2
PCDH10						■					protocadherin 10
POF1B						■					premature ovarian failure, 1B
PRRT3						■					proline-rich transmembrane protein 3
SH3BGRL						■			■		SH3 domain binding glutamic acid-rich protein like
SNAP29						■					synaptosomal-associated protein, 29kDa
TACSTD2						■					tumor-associated calcium signal transducer 2
TLE1						■					transducin-like enhancer of split 1 (E(sp1) homolog, Drosophila)
TMTC1						■					transmembrane and tetratricopeptide repeat containing 1
CTPS2							■				CTP synthase II
GLA							■			■	galactosidase, alpha
BASP1								■			brain abundant, membrane attached signal protein 1
NEDD9								■			neural precursor cell expressed, developmentally down-regulated 9
TACC1								■			transforming, acidic coiled-coil containing protein 1
ABLIM3									■		actin binding LIM protein family, member 3
ALG8									■		asparagine-linked glycosylation 8 homolog (S. cerevisiae, alpha-1,3-glucosyltransferase)
HEBP1									■		heme binding protein 1
MCF2L									■		MCF.2 cell line derived transforming sequence-like
STK39									■		serine threonine kinase 39 (STE20/SPS1 homolog, yeast)
WWOX									■		WW domain containing oxidoreductase
MANEA										■	mannosidase, endo-alpha
MCM6										■	MCM6 minichromosome maintenance deficient 6 (MIS5 homolog, S. pombe) (S. cerevisiae)

Figure S8. Kaplan-Meier survival analysis

Kaplan-Meier plot of survival outcome for ER positive breast cancer patients correlated with FOXM1 expression using the online tool KMplotter [11].

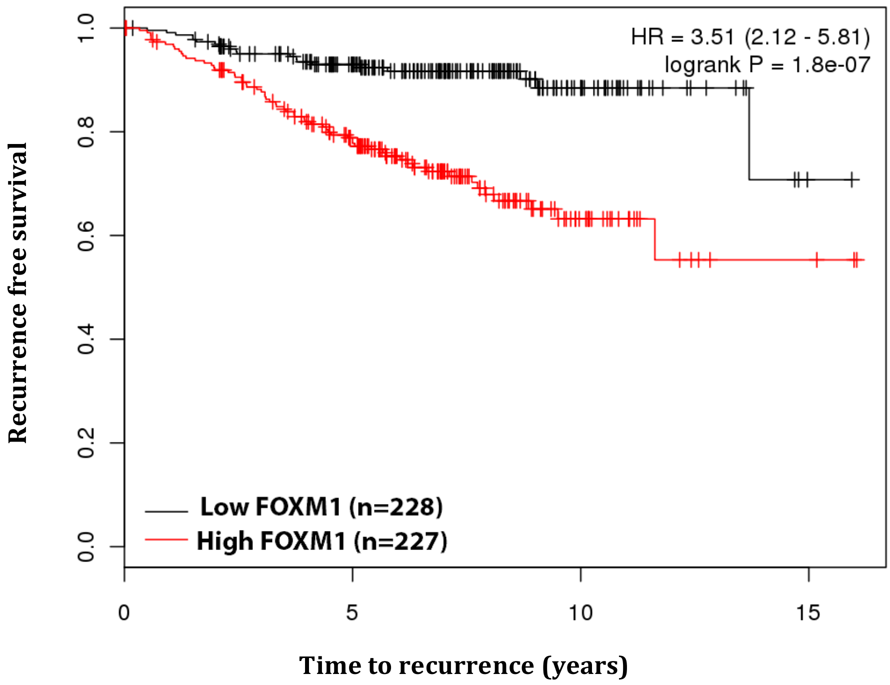

Figure S9. Clustering analysis with Wang breast cancer dataset

Clustering analysis of the patients in the Wang breast cancer dataset based on expression levels of the thiostrepton regulated genes (38 genes) separates the patients into 2 main groups (Low or High), which were used for generating a Kaplan-Meier survival plot.

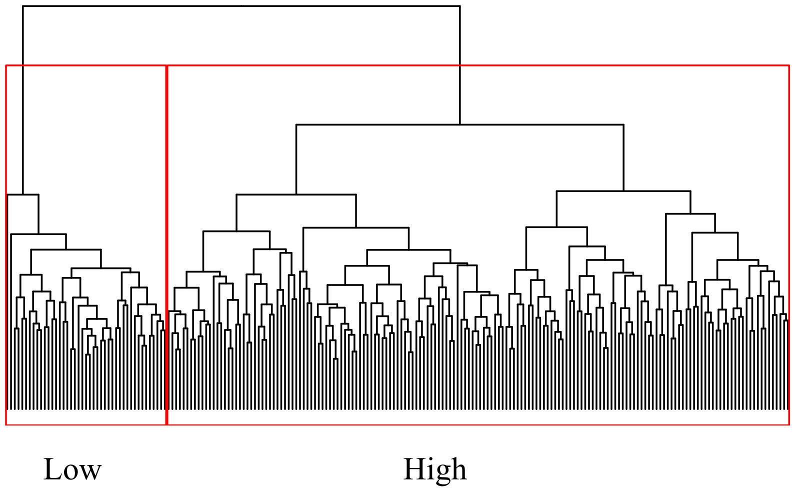

Figure S10. Heatmap and clustering analysis with Loi breast cancer dataset

The 38-gene (42 probes) signature associated with patient prognosis identified from the genes down-regulated by thiostrepton and with a FOXM1 binding site (+/-50kb TSS) in the Wang *et al* dataset were found to predict survival in an independent patient dataset [10]. (**A**) Heat map showing gene expression in the ER positive patients. Patients are grouped into good prognosis (non-relapsed, blue ribbon) and poor prognosis (relapsed, red ribbon). Gene expression is represented as light blue or red for patients with expression below or above the gene median, respectively. (**B**) Clustering of the patients by expression levels of these genes separates the patients into 2 main groups (Red= High, Blue=Low).

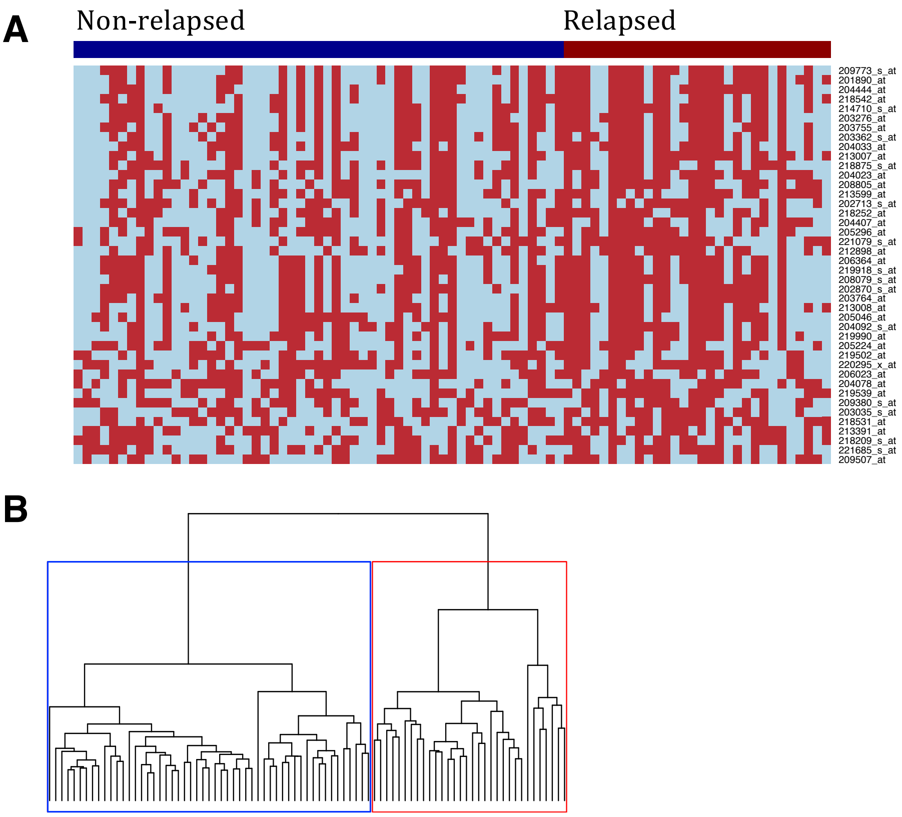

Figure S11. STRING protein interactions

STRING analysis of functional protein interactions for genes correlated with poor outcome in ER positive breast cancer. Genes were identified as down regulated following thiostrepton treatment of MCF7 cells and shown to have FOXM1 binding peak within 50kb TSS (ChIP-seq). High expression of these genes is significantly associated with poor prognosis in ER positive breast cancer study set (Wang *et al*). High confidence (score >0.7) interactions are shown.

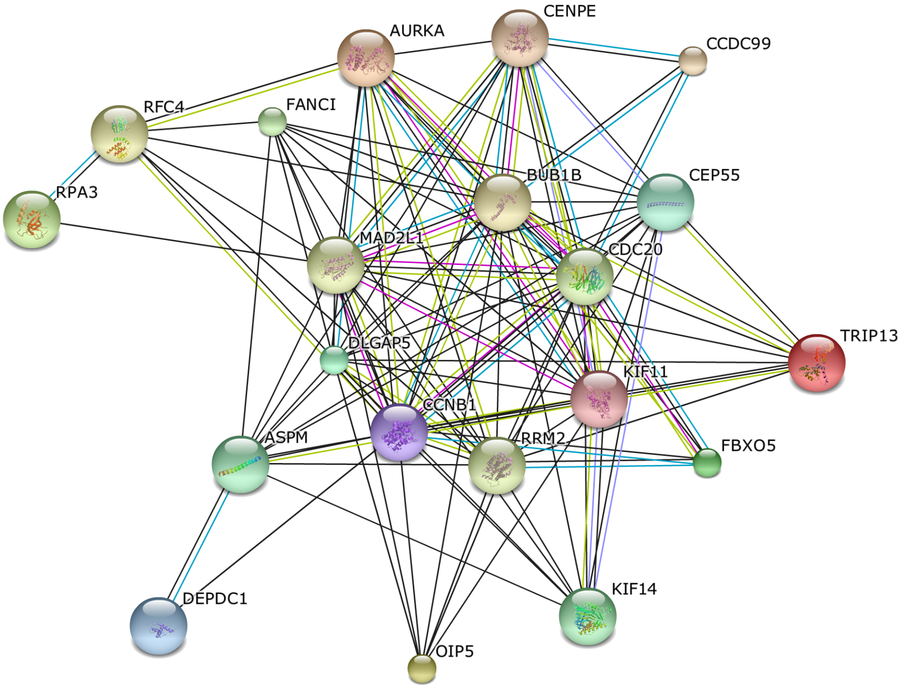

Supplement: Additional file 1 — Supplementary materials and methods, tables and figures as mentioned in the text. [file gb-2013-14-1-r6-S1.PDF]
